# Tracking Local Mechanical Impact in Heterogeneous Polymers with Direct Optical Imaging

**DOI:** 10.1002/anie.201809108

**Published:** 2018-10-09

**Authors:** Georgy A. Filonenko, Jody A. M. Lugger, Chong Liu, Ellen P. A. van Heeswijk, Marco M. R. M. Hendrix, Manuela Weber, Christian Müller, Emiel J. M. Hensen, Rint P. Sijbesma, Evgeny A. Pidko

**Affiliations:** ^1^ Inorganic Systems Engineering group Department of Chemical Engineering Delft University of Technology 2629 HZ Delft The Netherlands; ^2^ Institute for Complex Molecular Systems Eindhoven University of Technology 5600 MB Eindhoven The Netherlands; ^3^ Department of Chemical Engineering and Chemistry Eindhoven University of Technology 5600 MB Eindhoven The Netherlands; ^4^ Institut für Chemie und Biochemie Freie Universität Berlin 14195 Berlin Germany

**Keywords:** copper, luminescence, mechanical properties, molecular dynamics, polymers

## Abstract

Structural heterogeneity defines the properties of many functional polymers and it is often crucial for their performance and ability to withstand mechanical impact. Such heterogeneity, however, poses a tremendous challenge for characterization of these materials and limits our ability to design them rationally. Herein we present a practical methodology capable of resolving the complex mechanical behavior and tracking mechanical impact in discrete phases of segmented polyurethane—a typical example of a structurally complex polymer. Using direct optical imaging of photoluminescence produced by a small‐molecule organometallic mechano‐responsive sensor we observe in real time how polymer phases dissipate energy, restructure, and breakdown upon mechanical impact. Owing to its simplicity and robustness, this method has potential in describing the evolution of complex soft‐matter systems for which global characterization techniques fall short of providing molecular‐level insight.

Understanding the mechanisms that define mechanical stability of polymers is crucial for designing robust materials resistant to wear and failure. Although conventional macroscopic techniques such as rheology or dynamic mechanical thermal analysis (DMTA) may be sufficient to characterize most polymers, the macroscopic picture provided by these techniques often lacks molecular‐level resolution. This limitation is particularly valid for functional polymers that are typically non‐uniform and heterogeneous on multiple length scales. To understand properties of materials composed of several distinct or mixed phases a conceptually different characterization approach would be required.

A complex multi‐phase material at the core of this work, is a chain‐extended polyurethane (PU)—a copolymer composed of flexible polyether or polyester “soft” blocks and rigid “hard” blocks that form a hydrogen‐bonded crystalline phase (Figure 1).^1^ The ubiquity of PU in practical applications stems from the great variability of their properties, which can be achieved by tuning the chemical nature and relative content of different phases. Such diversity, however, comes at the expense of the drastically increased structural complexity of PUs. As a consequence, a local, phase‐ and component‐specific picture of their behavior under mechanical stress remains a topic of intense debate eight decades after the first PU was prepared.

**Figure 1:**
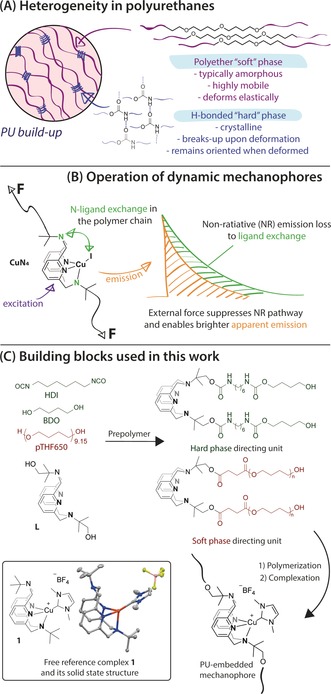
Structure and properties of PU phases (A), working principles of **CuN_4_** mechanophores (B) and building blocks used in this work together with the solid‐state structure of reference complex **1** (C).

Seeking to overcome the limitations of global characterization techniques lacking molecular‐level resolution, several force‐responsive molecular probes have been developed over the last decade. With few exceptions utilizing nanoparticle moieties,^2^ the majority of such probes are small organic fragments built into the polymer main chain. Upon exposure to external force, these organic moieties, often termed as mechanophores, can undergo mechanochemical activation^3^ producing a specific response, for example, luminescence^4^ or a change in absorbance and fluorescence characteristics.^5^, ^6^ Alternatively, a mechanical stimulus can alter the arrangement of fluorescent mechanophore within the polymer matrix, which results in a dramatic change of their emission properties.^7^ The vast majority of these mechanophores relies on transformations with no or limited reversibility. For this reason, common mechanophores can only probe the state of the polymer system before and after the mechanical impact while the dynamic evolution of the polymer system overtime remains beyond their reach.

Herein we address this challenge and develop a simple and straightforward methodology that allows the direct monitoring of the real‐lime evolution of discrete phases in a complex polymer system under mechanical stress. Instrumental to this work was the development of new robust organometallic mechanophores that combine high aerial stability and facile response with straightforward chemistry for their incorporation into distinct phases of polyurethanes. Relying on direct optical imaging as the primary analysis technique, the methodology holds great potential for general use in soft matter characterization.

The preparation of the new mechanophore and its selective incorporation in PUs was the first objective of our study. We based our mechanophore design on a new class of recently developed phosphorescent complexes that were shown to reversibly respond to mechanical stress (**CuN_4_**, Figure 1 b).^8^, ^9^ The mechanism governing their force‐induced response, depicted in Figure 1 b, is conceptually different from those found in common organic mechanophores.^9^ Instead of bond scission and related irreversible transformations, these copper halide complexes vary their photoluminescence (PL) via a force‐induced restriction of their intramolecular isomerization that represents a non‐radiative relaxation process. Slower isomerization, induced by the restriction on the polymer chain mobility under stress, results in brighter phosphorescence with higher quantum yields and longer PL lifetimes. Fast isomerization, that is typical for highly mobile polymer chains results in lower PL intensities. This response mechanism would be suitable to track force‐induced phenomena in PUs in real time but falls short of being practically useful due the poor air stability of the Cu^I^ halide complexes.

For practical reasons, the target stress‐responsive mechanophores based on the **CuN_4_** core should be robust enough to withstand continuous exposure to air and UV light. A common strategy for stabilizing Cu^I^ towards oxidation is the use of strong donor auxiliary ligands, for example, phosphines or N‐heterocyclic carbenes (NHC). To identify potential mechanophores we evaluated a series of Cu^I^ complexes with different NHC ligands using DFT calculations (S6 of Supporting Information) targeting complexes with the isomerization mechanism identical of that of previously described Cu halides. Specifically, we analyzed how mechanophore isomerization is influenced by substitution of the halide ligand in the parent halide **CuN_4_** by NHC ligands with different steric bulk. We identified one of the smallest known NHC ligands derived from N,N′‐dimethylimidazolium carboxylate (Figure 1) to be the suitable auxiliary ligand that would not alter mechanophore isomerization mechanism and therefore its force response. In line with predicted behavior, reference complex **1**, prepared to validate our analysis, indeed exhibits isomerization dynamics identical to that of **CuN_4_** and remains structurally similar to the parent complex according to the single crystal X‐ray diffraction data (see Figure 1 C and Section S5 of Supporting Information). This behavior, particularly important for mechanophore selection, suggests an isomerization mechanism of **1** is identical to that of parent halide mechanophores^9^ involving intermediate formation of 4‐coordinate Cu centers, thus, being associative. Finally, complex **1** is remarkably stable and withstands nearly a week long exposure to air in dichloromethane solution, making it suitable for further practical use as a mechanophore.

We further incorporated this phosphorescent motif into hard and soft phases of polyurethane samples. Inspired by the strategy reported by Braun and co‐workers^5^ we developed a one‐pot protocol targeting the macrocyclic ligand **L** (Figure 1) to prescribed locations. Namely, we pre‐formed two building blocks depicted in Figure 1 a “soft” one terminated with polyether groups (pTHF) and a “hard” one terminated with hexamethylene diisocyanate (HDI) extended with 1,4‐butanediol (BDO) to direct the mechanophore precursor to soft or hard PU domains, respectively. Respective PU samples were denoted as SP/HP for the type of PU phase hosting mechanophore and given the index “40” indicating the molar fraction of the BDO chain extender. NMR spectroscopy confirmed the incorporation of preloaded amounts of ligand **L** in all PUs and their identical composition regardless of the phase labelled (See Supporting Information). FTIR spectroscopy confirmed no impact of mechanophore incorporation on the hard phase of **SP40** samples and indicated a marginal increase in the relative content of disordered carbonyl groups in **HP40** further confirming that the labelling impacted specific synthetically targeted phases (See Figure S29 in the Supporting Information). Size exclusion chromatography indicated similar molecular weights of all samples ranging from 70 to 90 kDa with nearly identical polydispersity of 1.9–2.1. Finally, the polymer‐bound ligand **L** was complexed with Cu^I^ following the procedure developed for the synthesis of complex **1** to produce emissive polymers with ^1^H NMR spectra nearly identical to those of the reference complex **1** (See Section S3 of Supporting Information). The latter, importantly, confirms the identity of mechanophore embedded in the PU material and suggests no ligand exchange between mechanophore and polymer matrix taking effect.

Copper‐containing PU films fabricated by mold casting were photoluminescent and emitted at around 580 nm upon excitation with UV light. Importantly, the emission of PU films was identical to that of reference complex **1** in solution but not in the solid state. This confirms that mechanophore in the PU is well dispersed and not aggregated as the aggregates would produce emission similar to that of crystalline **1,** which is significantly blue shifted to approximately 555 nm. Films of **Cu‐HP** samples incorporating the mechanophore in the hard domain exhibit nearly three‐fold longer PL lifetimes compared to their soft phase labelled counterparts **Cu‐SP** suggesting that **Cu‐SP** indeed contains mechanophore in the phase with significantly higher chain mobility as expected when the mechanophore is embedded in the soft phase of PU.

Once discrete phases of PU samples were labelled with mechanophore, we focused on tracking the differences in force‐induced behavior of those phases. To do that, PU films were subjected to uniaxial extension with simultaneous detection of the photoluminescence intensity produced by these films. For tensile tests the isotropic PU films were pre‐stretched to 250 % of their initial length to prevent necking and associated changes in sample geometry during the imaging experiment.

To observe both deformation and stress relaxation kinetics we adopted the testing protocol (Figure 2) similar to that used by Braun^5^ and Boyce.^10^ Namely, samples were extended with a constant rate of 5 or 50 % L_0_ s^−1^ to produce a fixed (7.5 MPa) increase in stress at each loading step followed by a 5 second hold allowing for stress relaxation to be monitored. Afterwards, the loading/relaxation was repeated until the sample failed. Typical curves produced in these tests are depicted in Figure 2.

**Figure 2:**
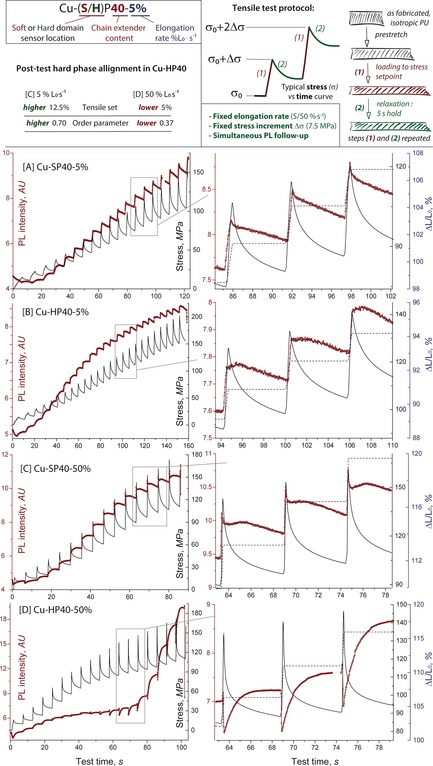
Tensile test method, hard phase alignment characterization (top) and experimental data (bottom, (A)–(D)). Stress (black lines) in uniaxial tension at two different elongation rates and corresponding PL response curves (brown dots). Expansions show PL, stress and strain profiles for three loading steps in the vicinity of 100 MPa region (curves in stress–strain coordinates can be found in the Supporting Information).

As expected, external stress applied to PUs reduces mechanophore isomerization rate and leads to an increase of the PL intensity during all tests. The evolution of PL response during stress relaxation step, however, depended strongly on the location of the phosphorescent probe and strain rates applied during the elongation. Slow deformation of the **Cu‐SP40** containing mechanophore in the soft phase produces an immediate increase of PL intensity that further decays when the sample is allowed to relax (Figure 2 A). Simultaneously with the decrease of the PL intensity, the global mechanical stress decreases. The drop on the PL intensity of the soft phase embedded mechanophore suggests that gradual redistribution of local stresses within the PU sample from the soft phase to the hard phase takes place during the relaxation stage. We therefore assume that even at low loading rates deformation of the soft phase is impacted by the deformation of the hard phase that accounts for decrease in the detected PL response.

Interestingly, mechanophore localized in the hard domain as in **Cu‐HP40** produces a different PL profile that contains a short plateau of approximately 1.2–1.8 s duration that precedes the PL decrease associated with the same stress redistribution phenomena that we observed in **Cu‐SP40**. Because the appearance of plateau was specific to the hard phase we assigned it to the reorientation of the hard phase along the elongation direction that was observed experimentally (See Figure 2 and Figure 3 and Supporting Information) and reported for other common PUs.^1^, ^11^ A common feature of slow deformation in either **HP** or **SP** samples is the immediate increase of PL intensity in response to deformation that indicates an immediate restriction of the chain mobility upon loading regardless of the mechanophore location.

Fast deformation of the hard phase labelled **Cu‐HP40** produces a dramatically different PL response. Upon deformation the PL intensity instantaneously drops while subsequent stress relaxation and associated decay of global mechanical stress is accompanied by a mono‐exponential increase of PL intensity (Figure 2 D). This sharply contrasts the behavior of the PL probe in **Cu‐SP40** at the same deformation rate (Figure 2 C) where no pronounced drop‐recovery behavior was observed.

Up to this point, our observations suggest that at high deformation rates the hard phase restructures differently with mechanophore becoming highly mobile upon mechanical impact and being slowly immobilized over time. Importantly, the extent of plastic deformation in **Cu‐HP40‐5/50** is negligible and the distinctly different hard phase behavior takes place within the same range of applied stresses and strains to those registered throughout all tests (see expansions in Figure 2). Taken together, these data suggest that observed PL response differences in **Cu‐HP40‐5/50** are specific to the hard phase of PU and stem solely from the elongation rate dependence. Having observed such a profound difference in the PL response for **Cu‐HP40** we further aimed at assigning these differences to physical events taking place in PUs under stress. This would ultimately allow linking the data provided by our methodology to the knowledge framework developed for PUs using conventional characterization techniques.

The major structural change occurring in PUs upon deformation is associated with the reorientation and segmentation of the crystalline hard phase.^1^, ^11^ Using linear dichroism IR spectroscopy we found that slow deformation of **Cu‐HP40** leads to a large increase of hard phase orientation along the stretching direction. The order parameter in that case increases from the initial value of 0.37 for pre‐stretched samples before tensile tests to approximately 0.7 (Figure 2, top). The increase of the order parameter is in line with the expected behavior of PUs, which are known to produce oriented morphologies upon deformation though the slow rotation of hard domains that orients them along the principal deformation axis.^11^ The observed mechanophore behavior in the slow deformation experiment is indeed consistent with this conventional deformation mechanism. The increase of PL intensity and the following slow decay can, therefore, be assigned to two distinct events in PUs upon deformation: 1) restriction of mobility of extended polymer chains (increase of PL) and 2) rotation of hard domains that allows for higher chain mobility as the stress relaxation takes effect (slow decrease of PL).

Aiming to identify the origin of a different PL response profile observed in the case of fast deformation we extended our structural studies to **Cu‐HP40‐50** sample. Interestingly, we found no apparent difference in the close range packing in **Cu‐HP40‐5/50** examined by wide angle X‐ray scattering (WAXS, see S7 in Supporting Information). IR spectroscopy, similarly indicated no detectible change in the overall hard phase composition in all **Cu‐HP40** samples as evidenced by identical absorbance in the characteristic regions for urethane NH and C=O vibrations (Figure 3 B and section S7 in Supporting Information). The hard phase alignment in **Cu‐HP40‐50**, however was found to be strikingly different from that of **Cu‐HP40‐5**. Namely, the fast deformation did not affect the hard phase order parameter that remained at the value of 0.37 after the tensile tests. This suggests that hard domains in PU were unable to rotate and re‐orient upon the fast deformation that resulted into a PU with an entirely different morphology. This notion is in part supported by atomic force microscopy data (Figure S28 in the Supporting Information) indicating a major restructuring of the PU surface as the PU samples are subjected to mechanical impact.

**Figure 3:**
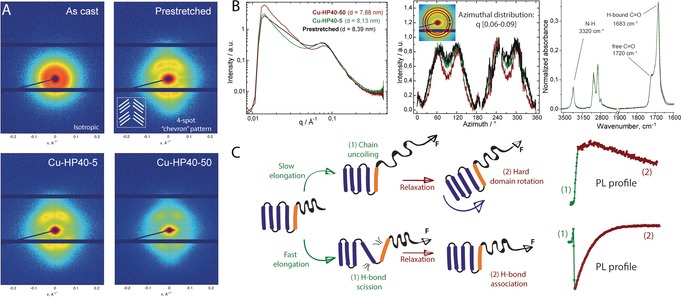
A) 2D SAXS data for top: isotropic and prestretched **Cu‐HP40** samples and bottom: of **Cu‐HP40‐5** subjected to slow elongation and **Cu‐HP40‐50** subjected fast elongation. Measurements performed on samples recovered after tensile elongation tests depicted in Figure 2. B) Total scattering intensity and azimuthal intensity distribution plots for scattering data depicted in panel (A) and IR spectroscopy data for these samples. C) Proposed deformation mechanism of **Cu‐HP40** at slow and fast elongation regimes assigned to observed PL profiles.

Further structural differences in **Cu‐HP40‐5/50** were revealed by the 2D‐SAXS analysis depicted in Figure 3 A. All samples after the pre‐stretch and tensile tests exhibited a typical 4‐spot pattern often referred to as “chevron pattern” common among HDI‐based PUs.^12^ This scattering pattern indicates the presence of two groups of hard domains tilted at identical angle with respect to the deformation direction as depicted in Figure 3 A. In contrast to the linear dichroism IR data suggesting the reorientation of the urethane functional groups in slow deformation experiments and the lack of thereof in fast deformation tests, SAXS data indicates that hard domains in **Cu‐HP40‐50** produced by the fast loading are actually more ordered macroscopically (See Figure 3 B). Namely, a more defined “chevron” pattern is revealed by the azimuthal scattering intensity distribution data and a detectible shift was observed for the broad peak in SAXS profile at ca. 0.08 Å^−1^ corresponding to the hard domain repeating unit. Altogether, these findings confirm that deformation rate dependence of the mechanophore response in **Cu‐HP40** indeed originates from differences in hard phase deformation mechanism.

Our data consistently links the differences in PL response to the development of distinct PU morphologies. Figure 3 C summarizes our proposed mechanism for their formation. In line with previous observations and our structural characterization data, slow deformation allows for the rotation of hard domains in PU and their alignment with the stretching direction. This deformation has no major effect on the hard phase morphology and preserves its macroscopic structure as confirmed by 2D‐SAXS. As the PL response in hard and soft phase of **Cu‐SP/HP40** was similar at low elongation rates, we assume that deformation is largely dominated by the soft phase uncoiling and subsequent relaxation due to hard phase rotation (Figure 3 C).

In contrast to this, the fast deformation regime is apparently destructive for the hard phase of the PU at least during the initial deformation step. This interpretation is supported by the sharp drop in the PL intensity during the fast loading in **Cu‐HP40‐50** that suggests that the mechanophore is mobilized upon the impact. This can proceed either via a pull‐out of individual hard segments containing the mechanophore or a partial breakdown of the hard phase crystallites. Importantly, regardless of which of these phenomena takes place, it does not induce hard domain rotation, suggesting that mechanical impact restructures the hard phase without allowing it to diffuse and reorient macroscopically. Based on these considerations, we come to conclusion that the hard phase deformation should involve the scission of weak hydrogen bonds between individual hard segments.

As the hydrogen bonds are non‐covalent, they can form and break reversibly,^13^ allowing fragmented hard domains to re‐associate when deformation stops. This behavior could indeed be responsible for the recovery of PL response observed during fast deformation experiments. Considering that the recovery of PL signal in **Cu‐HP40‐50** is strictly mono‐exponential we conclude that the PL response profile in fact describes a single process that is likely crystallization of the hard phase with an observed rate constant of 0.93±0.1 s^−1^ calculated from exponential PL recovery traces—a value corresponding to a process lifetime in the order of one second.

Although highly unusual to occur at room temperature, the hard phase crystallization is consistent with our experimental data—in PUs crystallization is a fast process with a time scale of multiple seconds at room temperature.^14^ Direct analysis of non‐isothermal crystallization kinetics of **Cu‐HP40** performed using DSC, places an estimated crystallization rate constant at 4.7 s^−1^ at 25 °C (S4 in Supporting Information)—a value similar to that observed in imaging experiments. Although the H‐bond scission in PUs likely impacts a minor fraction of the hard phase, our methodology appears to have sufficient sensitivity to produce a readily detectible response to these transient physical events that otherwise remain undetected by techniques lacking temporal or spatial resolution.

In conclusion, our work provides a direct observation of diverse and distinctly different phase behavior patterns within a single polymer family and offers an unprecedented insight into the mechanically induced evolution of complex polymer system. The response of robust mechanophores has been used as a diagnostic tool to identify the deformation mechanisms leading to the development of distinct polymer morphologies that can hardly be predicted using global characterization techniques. Since the response of new mechanophores originates at the molecular level, it allows us to describe force‐induced phenomena locally. At the same time, the non‐destructive nature of mechanophore action allows these dynamic phenomena to be tracked in real time. This methodology is efficient in dealing with the case of structural complexity in polyurethanes. We, therefore, see no limitation for it to become a common tool assisting materials scientists in resolving soft matter complexity in the near future.

## Experimental Section

Full synthesis and characterization data and testing methodology description can be found in Supporting. CCDC 1849321 contains the supplementary crystallographic data for this paper. These data can be obtained free of charge from The Cambridge Crystallographic Data Centre.

## Conflict of interest

The authors declare no conflict of interest.

## Supporting information

As a service to our authors and readers, this journal provides supporting information supplied by the authors. Such materials are peer reviewed and may be re‐organized for online delivery, but are not copy‐edited or typeset. Technical support issues arising from supporting information (other than missing files) should be addressed to the authors.

Supplementary
