# Supplementary material for: Tracking Local Mechanical Impact in Heterogeneous Polymers with Direct Optical Imaging
Source: Angew Chem Int Ed Engl. 2018 Oct 9;57(50):16385–90. doi: 10.1002/anie.201809108 (PMC6348422; doi:10.1002/anie.201809108)
Supplement: Supplementary file 1 — Supplementary [file ANIE-57-16385-s001.pdf]

Angewandte Chemie
Eine Zeitschrift der Gesellschaft Deutscher Chemiker

Supporting Information

Tracking Local Mechanical Impact in Heterogeneous Polymers with Direct Optical Imaging

Georgy A. Filonenko, Jody A. M. Lugger, Chong Liu, Ellen P. A. van Heeswijk, Marco M. R. M. Hendrix, Manuela Weber, Christian Müller, Emiel J. M. Hensen, Rint P. Sijbesma, and Evgeny A. Pidko*

anie_201809108_sm_miscellaneous_information.pdf

Contents

S1. General considerations 3
S2. Synthesis 6
S3. Supplementary emission characterization and tensile tests data 17
S4 Supplementary DSC data 21
S5 Crystallographic analysis details 24
S6 Details of DFT calculations 26
S7 Supplementary X-ray scattering, FTIR, DCS and AFM data 28

S1. General considerations

All manipulations unless stated otherwise were performed using Schlenk or glovebox techniques under dry argon atmosphere. Anhydrous solvents were dispensed from an MBRAUN solvent purification system and degassed prior to use. Anhydrous deuterated solvents were purchased from Eurisotop and stored over 4Å molecular sieves. Polytetrahydrofuran (pTHF) was supplied by Sigma and dried at 60°C for 2 hours at 0.1 mbar vacuum. All other chemicals unless noted otherwise were purchased from major commercial suppliers (TCI Europe and Sigma-Aldrich) and used without purification.

Instrumentation and methods:

NMR spectra were measured on Bruker 400 Avance II, and Agilent 400-MR DD2 spectrometers. Electrospray Ionization Mass Spectrometry (ESI-MS) measurements were performed on a Thermo Scientific Fleet apparatus using direct infusion mode. Gel permeation chromatography (GPC) measurements were performed using HPLC setup equipped with Shodex KD-804 column operating at 40°C with DMF/LiBr (100mM) as the mobile phase supplied at 1 mL/min flow rate. Quantification was performed using refractive index detector, the apparatus was calibrated using PEO/PEG standards supplied by Sigma-Aldrich. Differential Scanning Calorimetry measurements were performed in crimped Al sample pans using a TA Q1000 DSC apparatus operating at 10 °C/min ramp rate in dry nitrogen. Polymer samples were used as prepared in ca. 10 mg amount. Samples were cooled to the minimal test temperature and the first heating cycle was discarded with subsequent cycles used for analysis. Non-isothermal crystallization kinetics was analysed using DSC curves registered using Perkin-Elmer DSC 7 apparatus at varied cooling rates. Full methodological description is given below in section **S4**.

X-ray scattering measurements were performed on a Ganesha lab instrument equipped with a Genix-Cu ultra-low divergence source producing X-ray photons with a wavelength of 0.154 nm and a flux of $1x10^8$ photons s^{-1}. Diffraction patterns were collected using a Pilatus 300K silicon pixel detector with 487 x 619 pixels of 172 μm^2 in size, placed at a sample to detector distance of 91 mm (wide angle, WAXS), or 500 mm (medium angle, MAXS). On the obtained diffraction patterns an azimuthal integration was performed, using SAXSGUI software, to calculate the intensity against the scattering vector q, where $q=(4\pi/\lambda)\sin\vartheta$ (ϑ is the angle of incidence and λ is the wavelength). The beam centre and the q-range were calibrated using silver behenate ($d_{(100)}$ = 1.076 nm^{-1}; 5.839 nm), as a reference. The $d_{(300)}$ was used for calibration.

Tensile test methodology:

Measurements were performed using Zwick Z100 machine with 100 N load cell. Polymer samples were cast from dichloromethane/methanol (10/1 vol/vol) solutions (160-200 mg material in 4 mL) maintained at 26°C in a PTFE mould by slow solvent evaporation at the same temperature and cut to rectangles of 5 mm width.

IMPORTANT NOTE: As reported previously[1] no PL change was observed in PUs at low deformations below the yield point associated with low stress levels of ca 5 MPa. The samples before the stress relaxation tests were, therefore, extended to 250% L_0 before the measurements to prevent yielding and necking during the test that would hamper accurate imaging. Prestretched samples were allowed to relax before clamping to the tensile tester fixtures. Prestretching has mainly plastic character and results in a 123-125 % tensile set that corresponds to a permanent increase in length from L_0 to 223-225% L_0. The length prestretched samples is considered initial length (L_0) in all tensile tests.

Prestretched samples were further clamped and loaded in a stepwise manner. Namely, each loading step performed either at 5 or 50 %L_0/s elongation rate was programmed to produce a fixed increase of peak stress value of 7.5 MPa. Once the set stress value was reached, the elongation was stopped and the sample was allowed to relax for 5 seconds before next loading step was performed.

PL intensity during the tensile tests was recorded using Thorlabs DCC3260C CMOS camera fitted with the FEL500 edgepass filter (OD>6) to block the UV light used for excitation (365 nm LED, ML365L2 from Thorlabs operating at 300-400 mA) in complete darkness. Video was captured at 40 fps rate and resulting image stacks were processed in ImageJ using Time Series Analyser plugin to obtain the average intensity of light emitted from the centre of the sample. The resulting intensities were plotted vs the frame number and rescaled to the time domain. Scaling was necessary to account for the dropped frames and 0.1-0.2 s delay caused by recording multiple video files during the single tensile tests requiring higher acquisition times.

Photophysical characterization

The **photoluminescence measurements** were performed using Edinburgh Instruments fluorimeter with excitation set at 400 nm.

Photoluminescence lifetime measurements were performed using Edinburgh Instruments Lifespec apparatus with excitation at 404 nm by pulsed laser light source. Emitted light was collected at the emission maxima determined previously for specific samples. Polymer films were mounted as cast, solid crystalline complex **1** was immersed into fluorinated oil and sandwiched between two quartz slides and solutions of complex **1** were sealed under Ar in borosilicate glass vials.

FTIR spectra were recorded using Bruker Alpha II spectrometer in attenuated total reflectance mode in the mid-IR range (4000-400 cm^{-1}). Dichroism measurements were performed on a Varian 670 IR spectrometer equipped with a slide-on ATR crystal (Ge) and a rotatable Infrared Polarizer in a Varian 610 microscopy setup over a range of 4000-650 cm-1 with a spectral resolution of 4 cm^{-1} and 100 scans per spectrum. Identical absorbance data was obtained for triplicate analyses of PU samples performed at three different spots within ca. 5 mm long section around the sample centre. Dichroic ratios were measured for C=O stretching peak at 1685 cm^{-1} that provided the most accurate absorbance readings. Dichroic ratios were converted to order parameters as described previously.[2, 3]

Atomic Force Microscopy (AFM) was used to characterize surface morphology of all samples. Characterization was performed with a NT-MDT NTegra Aura in a semi-contact, tapping, topography mode . The height as well as the phase images were obtained using NT-MDT NSG01 probes and Asylum Research AC240TS probes. The NSG01 probes have a typical resonance frequency of 150kHz and a spring constant of 5.5N/m with a tip radius of 10nm. The AC240TS probes have a typical resonance frequency of 70kHz and a spring constant of 2N/m with a tip radius of 7nm. The resulting images were flatten using a first order polynomial for compensating any sample slope. Images are shown below in section S7.

S2. Synthesis

Reference complex 1:

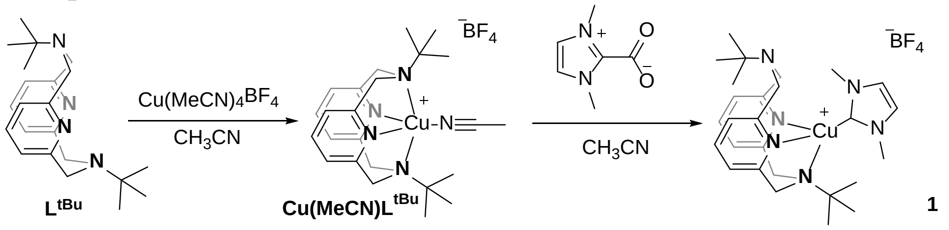

Complex **1** was prepared from **Cu(MeCN)L^{tBu}** generated via a published procedure[4] from **L^{tBu}** [5] and Cu(MeCN$_4$)BF$_4$. In a typical procedure 201.5 mg of **Cu(MeCN)L^{tBu}** (0.37 mmol) dissolved in 2 mL acetonitrile were treated with 54.4 mg dimethylimidazolium carboxylate (1.05 eq., 0.388 mmol) at room temperature. After stirring for 1 hour the carboxylate precursor dissolves and the colour of solution changes from bright orange to pale yellow. At this point reaction is complete as indicated by ^{1}H NMR and the product can be worked up by adding 5-10 mL diethyl ether, filtering the resulting suspension and placing it for 2 hours to the freezer at -18 °C. After cooling the yellow microcrystalline powder is recovered by decantation of the pale yellow mother liquor and washing with two 10 mL portions of ether with subsequent drying under vacuum. This furnishes 145 mg (Yield 65%) material that was confirmed to contain no impurities by ^{13}C NMR and CHN microanalysis. Samples for single crystal X-ray diffraction studies were grown from acetone/benzene mixture by slow vapour diffusion.

The synthesis of **1** was repeated in duplicates in dichloromethane and tetrahydrofuran solvents to provide similar yields. In these cases the addition of small amount of acetonitrile (typically 10 drops) was found to be critical to prevent the disproportionation of the starting Cu complex before NHC ligand complexation could take place. Variations of solvent type had no significant impact and yields of resulting product.

Complex **1** is stable as solid for at least a month in air. Dichloromethane solutions of **1** remain intact for at least 5 days upon exposure to air as evidenced by ^{1}H NMR (See Figure S4 below).

Due to the highly dynamic nature of the complex, NMR spectra were measured at -30 °C for accurate assignments.

^{1}H NMR (400 MHz, CD$_2$Cl$_2$, -30°C) δ 7.35 (t, $^{3}J_{HH}$ = 7.7 Hz, 2H, *p*-Py-C***H***), 7.03 (s, 2H, imi C**H**), 6.92 (d, $^{3}J_{HH}$ = 7.8 Hz, 2H, *m*-Py-C***H***), 6.79 (d, $^{3}J_{HH}$ = 7.5 Hz, 2H, *m*-Py-C***H***), 4.69 (d, $^{2}J_{HH}$ = 14.9 Hz, 2H, Py-C***H***$_2$-), 4.38 (d, $^{2}J_{HH}$ = 12.6 Hz, 2H, Py-C***H***$_2$-), 3.85 (d, $^{2}J_{HH}$ =

12.6 Hz, 2H, Py-C$\boldsymbol{H}_2$-), 3.79 (s, 6H, imi-C$\boldsymbol{H}_3$), 3.55 (d, $^2J_{HH}$ = 15.0 Hz, 2H, Py-C$\boldsymbol{H}_2$-), 1.39 and 1.18 (s, total 18H, ^{t}Bu-C$\boldsymbol{H}_3$).

^{13}C NMR (101 MHz, CD_2Cl_2, -30°C) δ 159.75 (s, Py-C_{quart}), 155.41 (s, Py-C_{quart}), 137.27 (s, Py-C_{para}), 124.13 (s, Py-C_{meta}), 121.40 (s, Py-C_{meta}), 120.93 (s, ***C***H_{imi}), 58.92 (s, overlap of Py-C$\boldsymbol{H}_2$-), 58.70 and 56.49 (both s, -***C***-$(CH_3)_3$), 37.60 (Imi-***C***H_3), 30.49 (broad s) and 27.37 (s) both -C-(***C***$H_3)_3$. The resonance of quaternary carbon of the NHC ligand was not observed due to low solubility of the compound and influence of chemical exchange.

EA: Calcd. (Found) $C_{27}H_{40}N_6B_1F_4Cu_1$ – C 54.14 (53.99); H 6.73 (6.69); N 14.03 (14.20)

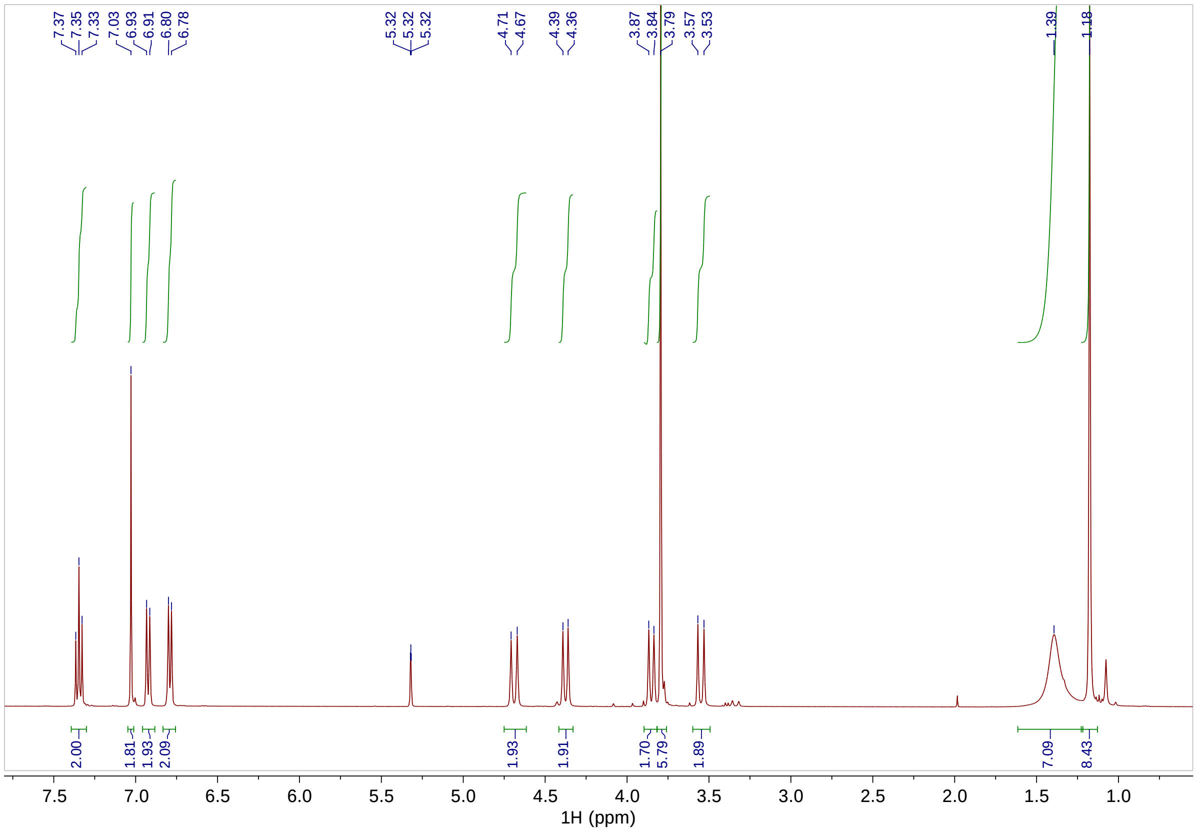

Figure S1. ^{1}H NMR spectrum of complex **1** in CD_2Cl_2 at -30 °C.

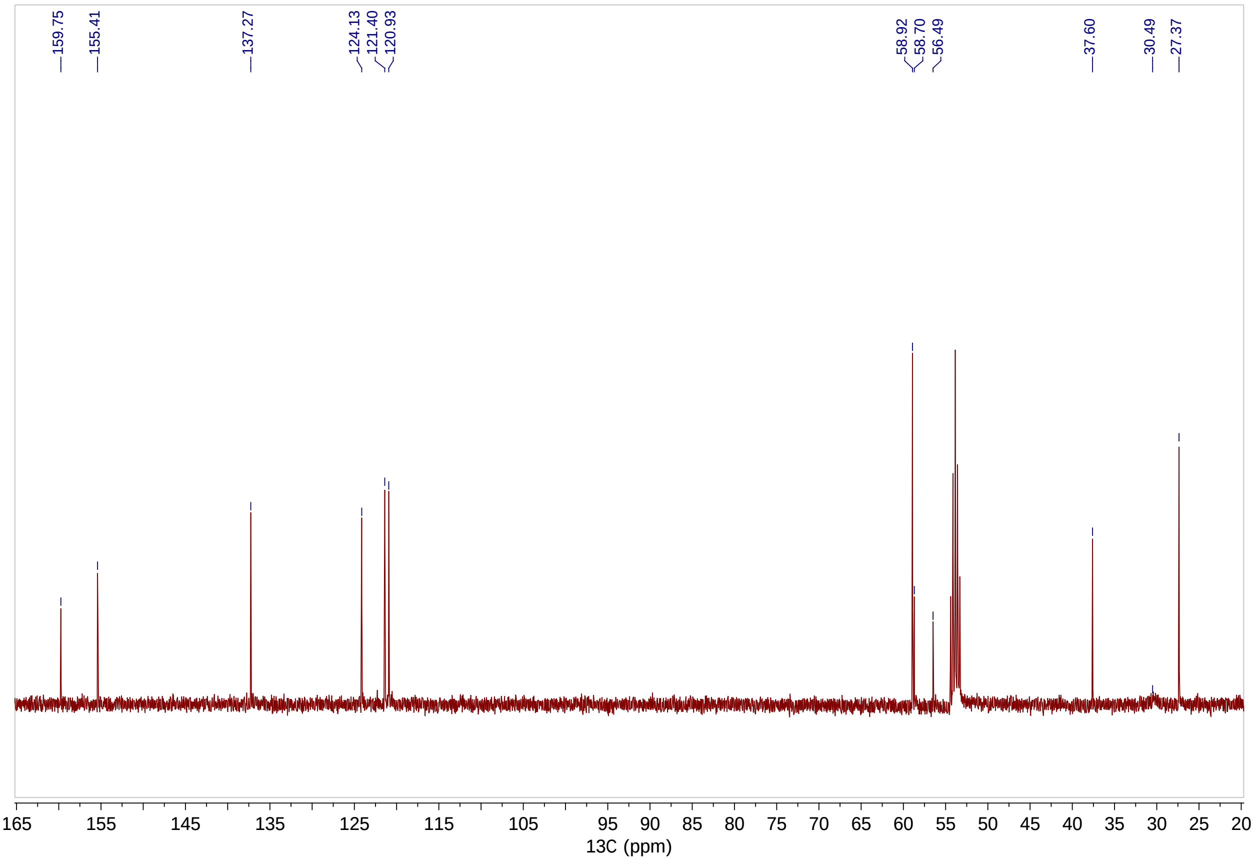

Figure S2. ^{13}C NMR spectrum of complex **1** in CD_2Cl_2 at -30 °C.

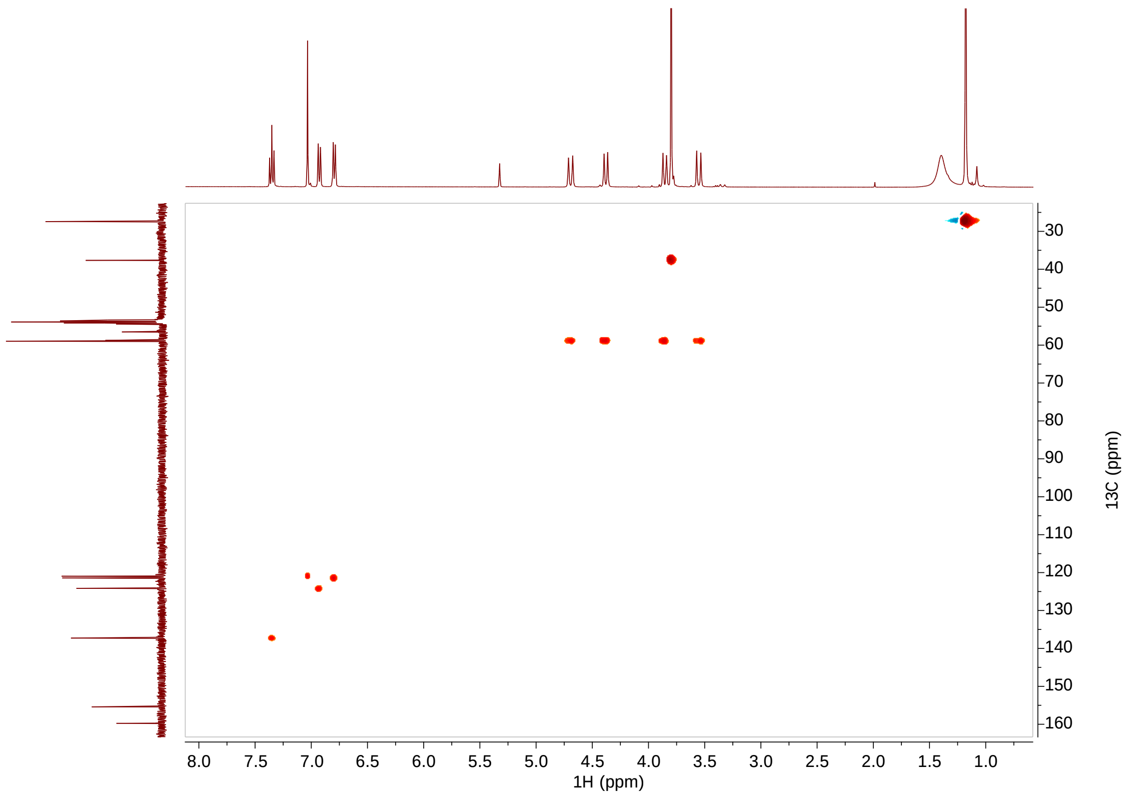

Figure S3. gHMQC spectrum of complex **1** in CD_2Cl_2 at -30 °C.

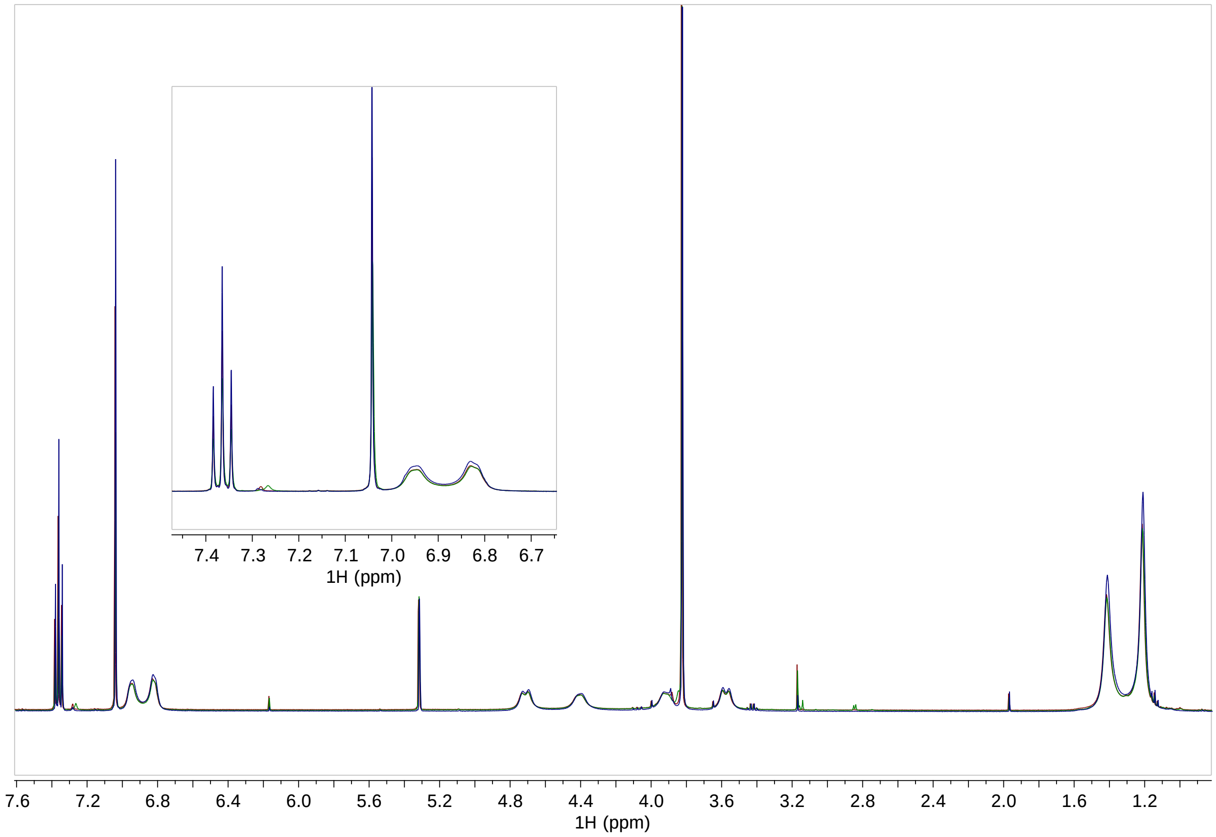

Figure S4. ^{1}H NMR spectrum of complex **1** in CD_2Cl_2 at 23 °C under Ar (blue curve) and the same sample exposed to air for 1 day (red line) and 5 days (green line) indicating the stability of **1** in solution.

Polyurethane synthesis:

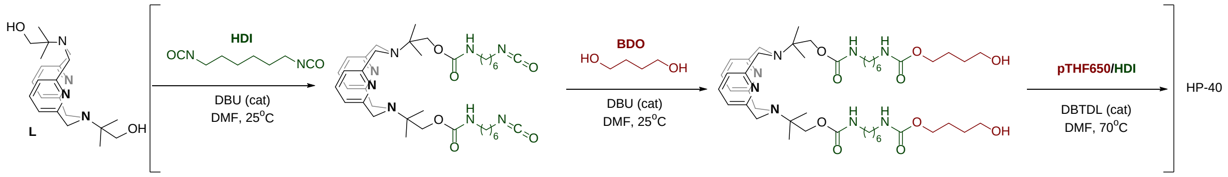

Hard block labelled HP-40:

Ligand **L**[1] (48 mg, 0.125 mmol) was taken up in 0.5 mL DMF and added to a DMF solution containing a drop of DBU and 315.37mg HDI (1.875 mmol). The capping reaction was allowed to proceed for 30 minutes until no free ligand could be observed by ESI-MS. At this point 1,4,-butanediol (326.68 mg, 3.625 mmol) in 5 mL DMF was added to the reaction mixture followed by a drop of DBTDL catalyst to terminate all isocyanate units with chain extender. After 30 minutes capping is complete as evidenced by the presence of a peak at m/z = 901 a.m.u. corresponding to BDO-HDI-L-HDI-BDO units detected by ESI-MS. The remainder of HDI (1156.37 mg, 6.875 mmol) and $pTHF_{650}$ (3250 mg, 5 mmol) were added afterwards followed by the drop of DBTDL catalyst and reaction mixture was heated at 70°C for 30 minutes. Over this time the solution became viscous and was quenched by addition of 1 mL methanol and poured into ether (300 mL) to precipitate white polymer product. Solvent was decanted and solid product was soaked in ether for 5 hours and dried under vacuum to yield 4.4 g of polymer HP-40 (ca 87% recovery). Ligand incorporation and content was checked by ^{1}H NMR.

HP-40: GPC (DMF/0.1M LiBr): M_w = 96.1 kDa; PDI = 2.12

NOTE: attempted preparation of the polymer in this protocol using dichloromethane or THF solvents fails due to the rapid formation of the white precipitate, presumably BDO-HDI oligomers. Any attempt to carry out further transformation in the presence of this solid did not lead to any polymerized product.

Soft phase labelled SP-40:

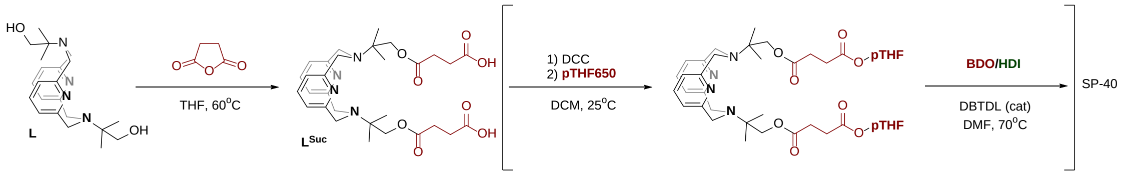

L^{Suc}: Ligand **L** (385 mg, 1 mmol) was taken up in 5 mL THF and treated with 200 mg succinic anhydride (2 mmol). Supension was incubated at 60°C overnight and evaporated to dryness to give crude **L^{Suc}** as white solid that was used without additional purification. NMR and ESI-MS data for **L^{Suc}** is given below. Further polymerization was performed on the same scale as for HP-40 in the following way:

Step 1) Capping of **L^{Suc}** was done following the adaptation of published protocol of Moore and Stupp.[6] **L^{Suc}** (73.1 mg, 0.125 mmol) was taken up in 1 mL dichloromethane and treated with DPTS (see Ref 6 for preparation; 36 mg, 1 eq) and DCC (77.4 mg, 3 eq.) and excess $pTHF_{650}$ (585 mg, 0.9 mmol). The mixture was stirred at room temperature for 1 hour to produce the white DCU urea precipitate that was filtered off before reaction was continued.

IMPORTANT NOTE: ^{1}H NMR control is recommended to confirm full conversion of **L^{Suc}** and completion of esterification. ESI-MS can also be used to verify the presence of series of pTHF capped **L^{Suc}** units and the absence of the starting material.
The use of THF or DMF solvent for the esterification step or/and lower amounts of DPTS catalyst leads to incomplete conversion and isomerization of the DCC activated acids. This prevents further polymerization and should be avoided.

Step 2) The filtrate containing pTHF-capped **L** was transferred to 40 mL DMF and remainder of HDI (1450.7 mg, 8.75 mmol) was added followed by a drop of DBTDL, 326.7 mg 1,4-butanediol (3.625 mmol) and 2665 mg pTHF (4.1 mmol for a total of 5 mmol pTHF). The reaction mixture was set at 70°C and allowed to polymerize for 30 minutes as described for HP-40 with the same workup.

Due to the high basicity, ligand **L** was found to scavenge the PTSA from the DPTS catalyst used in equimolar amount. This can be traced by the shift of **L** resonances in ^{1}H NMR to a lower field typically observed for N_4 macrocycles (See Figure S6b). To remove the PTSA moiety from the polymer, the product was stirred in acetonitrile (100 mL) containing 5

mL trimethylamine. The washing was repeated twice until ^{1}H NMR was consistent with the presence of free neutral **L** unit. Yield 4.2 g (83%)

SP-40: GPC (DMF/0.1M LiBr): M_w = 67.8 kDa; PDI = 1.95

Reference PU containing no mechanophore was prepared in a similar way. In 35 mL DMF the HDI (1450.7 mg, 8.75 mmol) treated with a drop of DBTDL and 326.7 mg 1,4-butanediol (3.625 mmol) was slowly added followed immediately by solution of 3331.2 mg of pTHF650 (5.125 mmol) in 10 mL DMF. The reaction mixture was set at 70°C and allowed to polymerize for 45 minutes as described for HP-40 with the same workup.

Ref PU: GPC (DMF/0.1M LiBr): M_w = 135.2 kDa; PDI = 1.84

See GPS curves, FTIR data and thermal behaviour characterization in sections below.

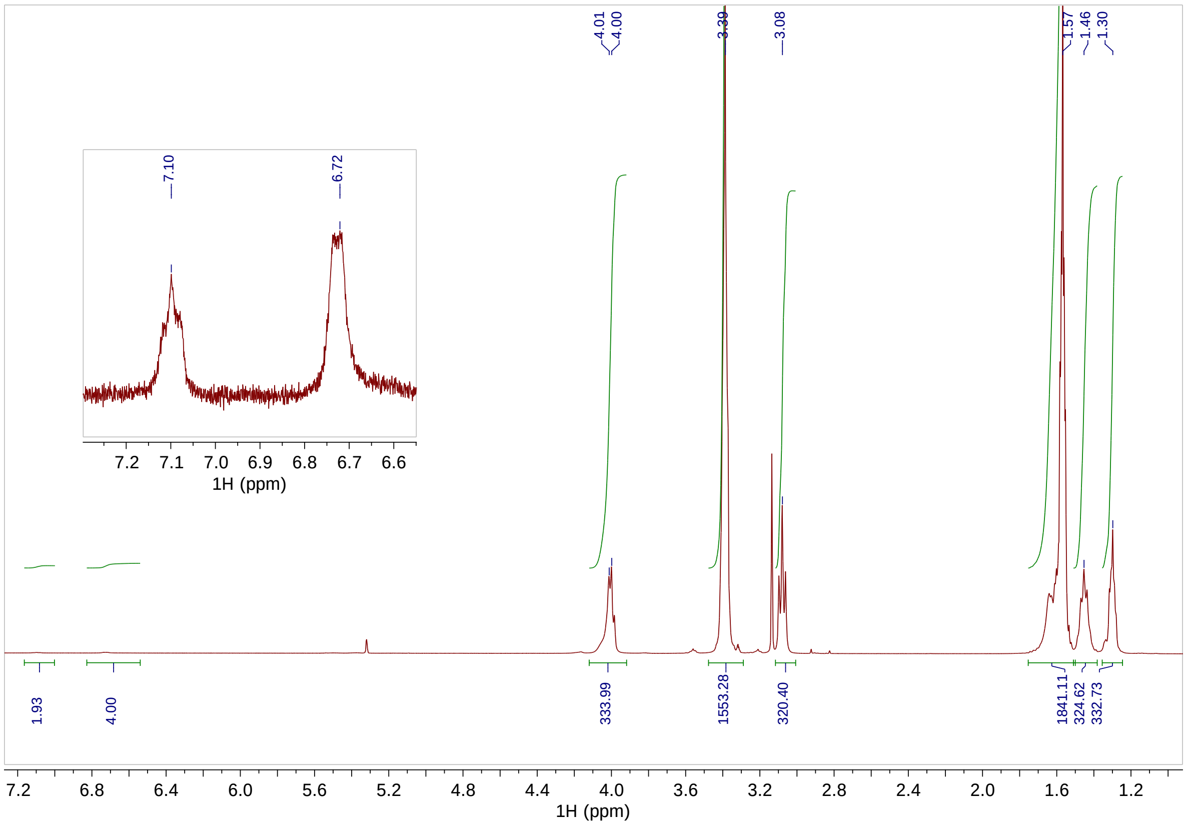

Figure S5. ^{1}H NMR spectrum of **HP-40** in CD_2Cl_2/MeOD (10/1) at 23 °C. Insert shows aromatic region where resonances of incorporated **L1** are visible in ca 1/80 ratio to HDI resonances vs preloaded 1/70 ratio.

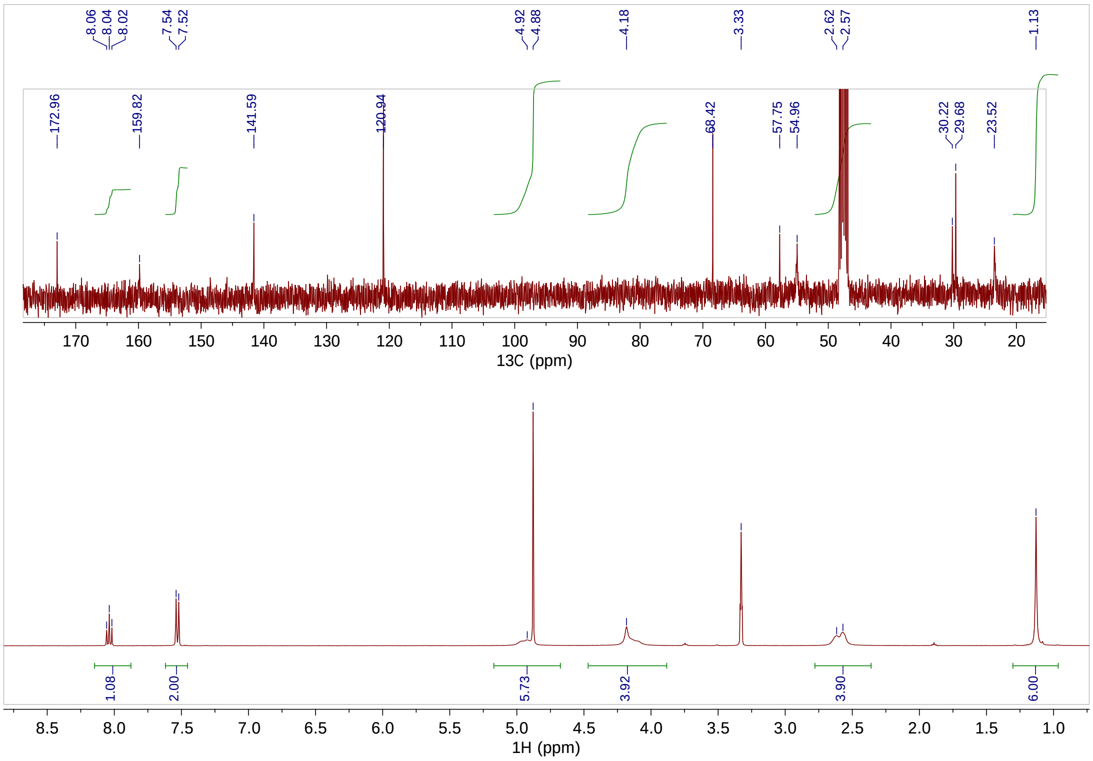

Figure S6a. ^{1}H NMR spectrum of $\mathbf{L}^{\mathbf{Suc}}$ in MeOD at 23 °C. Insert shows ^{13}C spectrum.

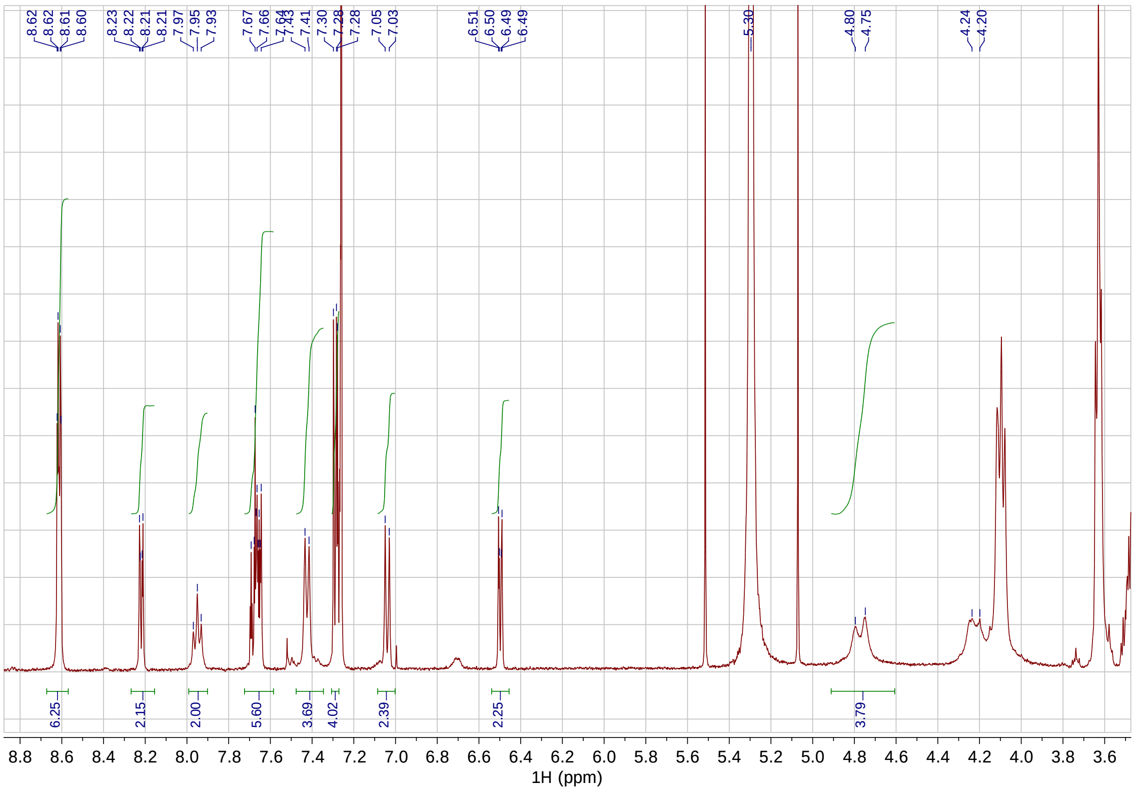

Figure S6b. ^{1}H NMR spectrum of crude reaction mixture ($\mathbf{L}^{\mathbf{Suc}}$ extended with pTHF units) after step 1 and before polymerization to SP-40 in CD_3Cl at 23 °C. Note the low field shift of aromatic resonances in **L** (7.95 and 7.42 ppm) and the inequivalency of methylene units (4.77 and 4.22 ppm) indicating protonation of the N_4 macrocycle.

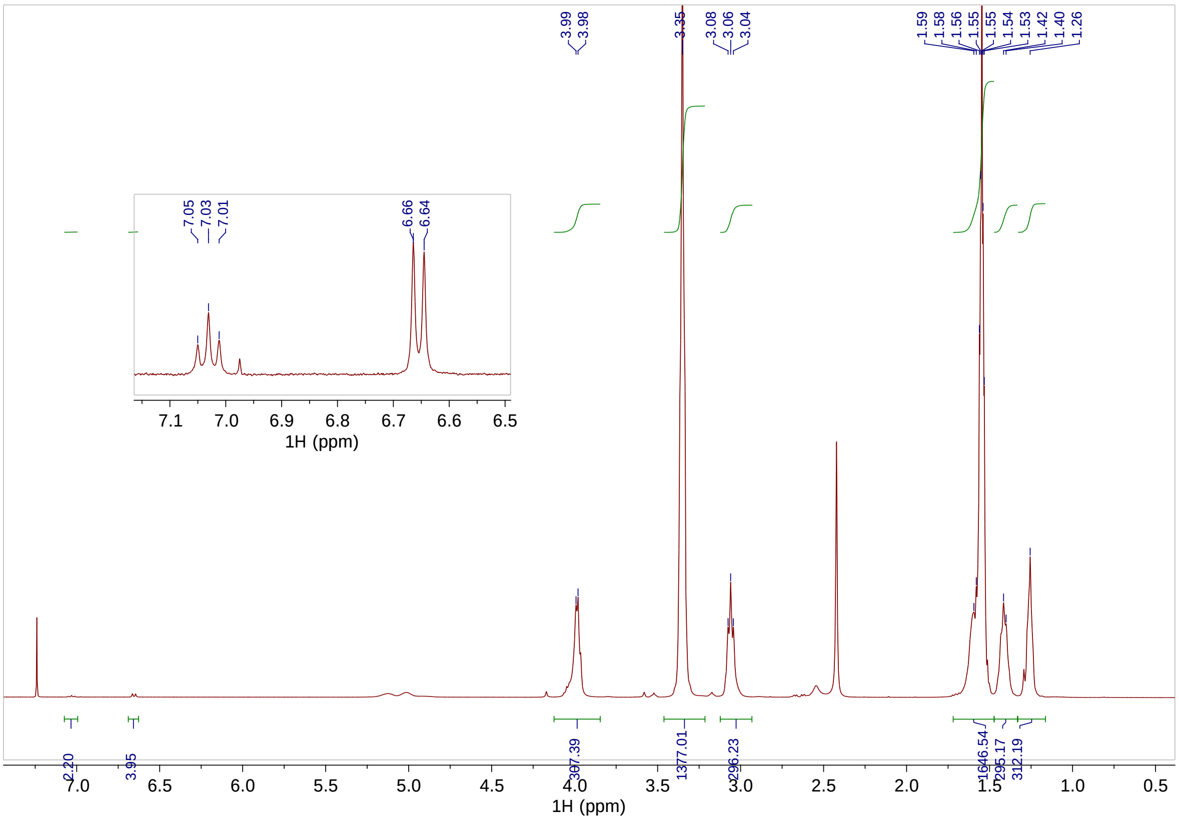

Figure S7. ^{1}H NMR spectrum of **SP-40** in $CDCl_3$/MeOD (10/1) at 23 °C. Insert shows aromatic region where resonances of incorporated **L1** are visible in ca 1/76 ratio to HDI resonances vs preloaded 1/70 ratio.

Polymer metallation procedure:

In a typical procedure 600 mg of polymer material (**L** content ca. 25 µmol/g polymer; 15 µmol) was taken up in 10 mL acetonitrile. To a stirred suspension (PU does not dissolve in acetonitrile, therefore care should be taken to disperse PU material as fine as possible typically by cutting with blade) an excess of copper precursor $Cu(MeCN)_4BF_4$ (8 mg, 25,5 µmol) was added to produce immediate colour change of the white polymer chunks to orange. Suspension was allowed to stir for 30 minutes to complete the formation of acetonitrile complex and was further treated with dimethylimidazolium carboxylate (3.6 mg; 25,7 µmol) at room temperature for 4 hours. Within this time polymer material turns pale yellow. Liquids are further decanted and polymer is washed with two portions of 5 mL of acetonitrile (1 hour tumbling) to remove traces of unreacted NHC ligand precursor and unbound copper complex. The sample is then washed with two portions of ether (10 mL, 1 hour each exposure) and dried under vacuum to yield metallated polymer. Formation of the target complex in all PUs was validated using ^1H NMR.

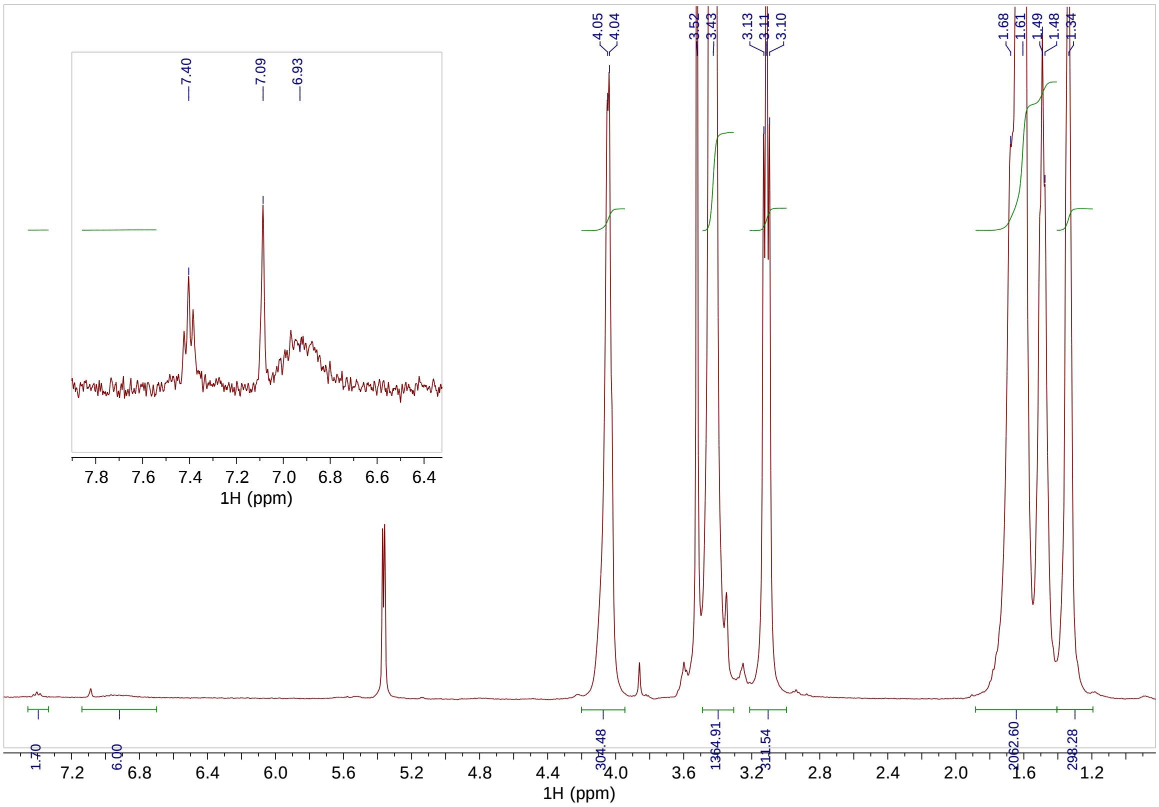

Figure S8. ^1H NMR spectrum of **Cu-HP-40** in CD_2Cl_2/MeOD (10/1) at 23 °C. Insert shows aromatic region with resonances of formed Cu complex similar to those of the reference complex **1**.

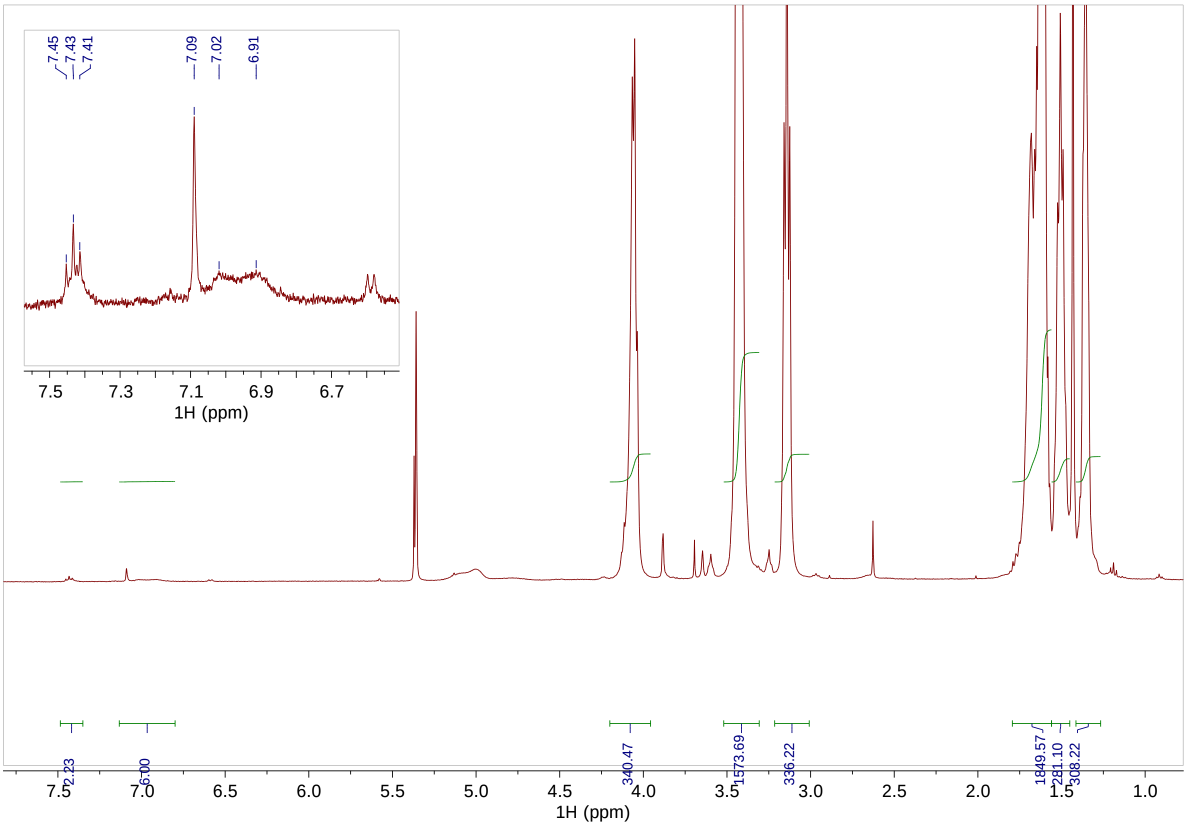

Figure S9. ^{1}H NMR spectrum of **Cu-SP-40** in CD_2Cl_2/MeOD (10/1) at 23 °C. Insert shows aromatic region with resonances of formed Cu complex similar to those of the reference complex **1**. Small resonance of free ligand was observed at 6.59 ppm.

S3. Supplementary emission characterization and tensile tests data

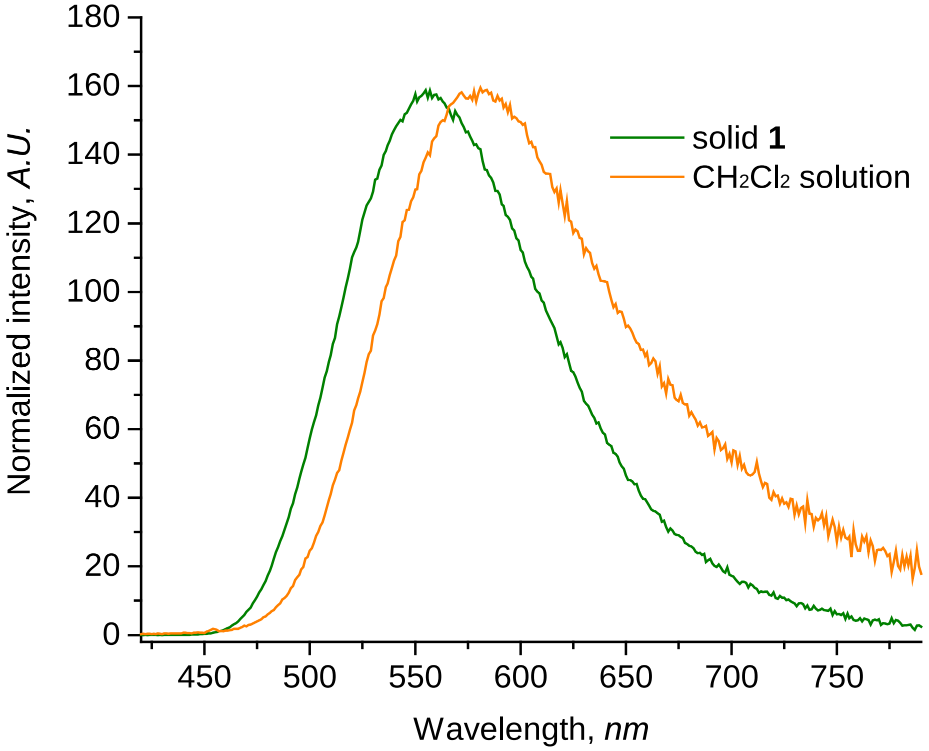

Figure S10. Emission spectra of complex **1** in solid state and dichloromethane solution (1 mg/mL) upon excitation at 400 nm. Note the blue shift of the emission profile of the solid complex.

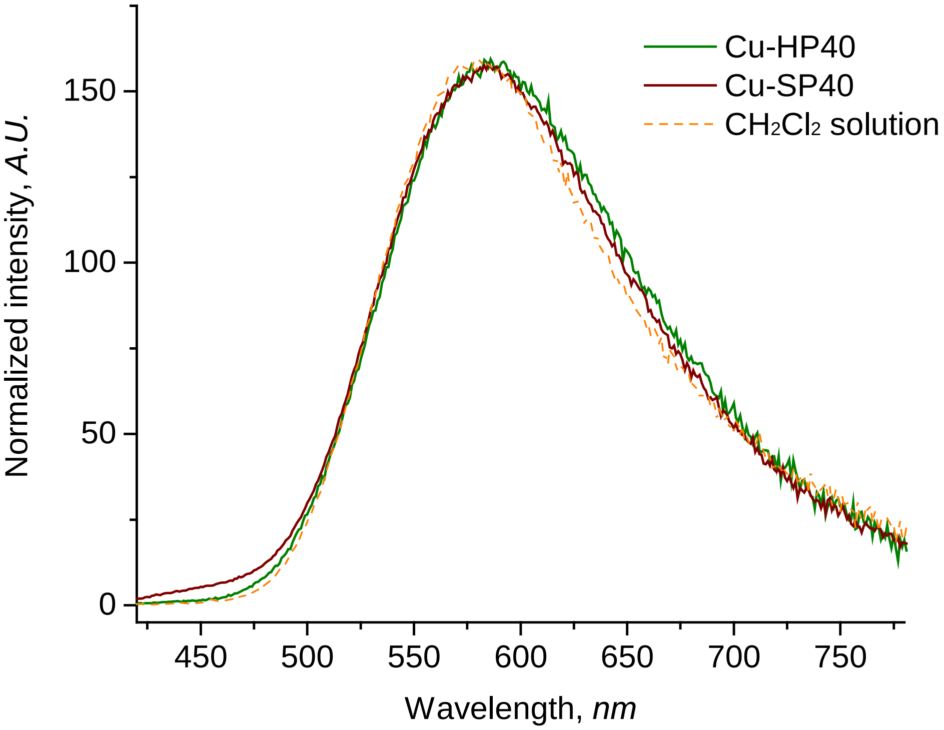

Figure S11. Emission spectra of Cu-HP/SP40 polymers upon excitation at 400 nm.

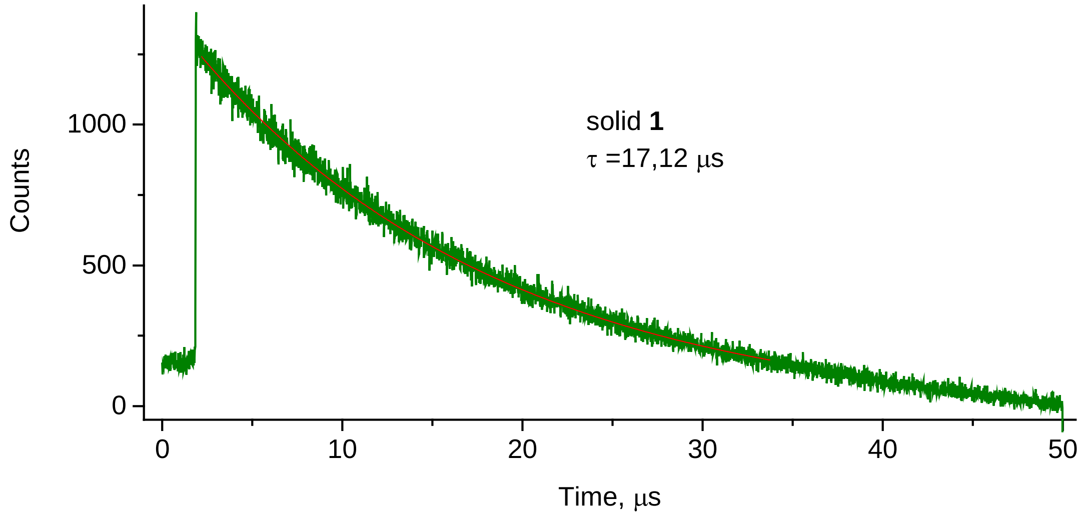

Figure S12. Photoluminescence decay curve of solid complex **1** measured at 540 nm upon excitation at 404 nm. Red line is the single exponential fit curve given for reference. Calculated lifetime indicated on the graph.

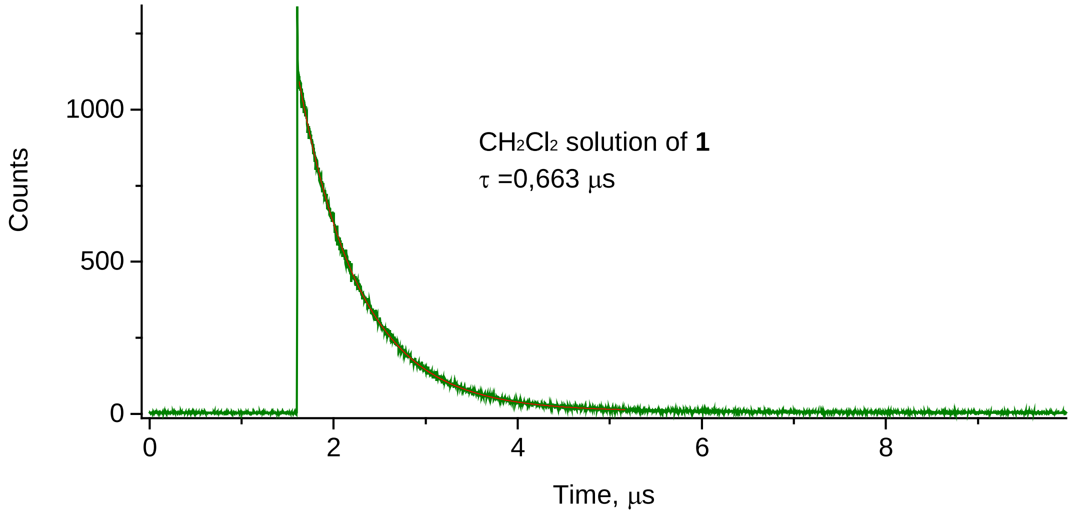

Figure S13. Photoluminescence decay curve of dichloromethane solution of complex **1** at concentration of 1 mg/mL measured at 560 nm upon excitation at 404 nm. Red line is the single exponential fit curve given for reference. Calculated lifetime indicated on the graph.

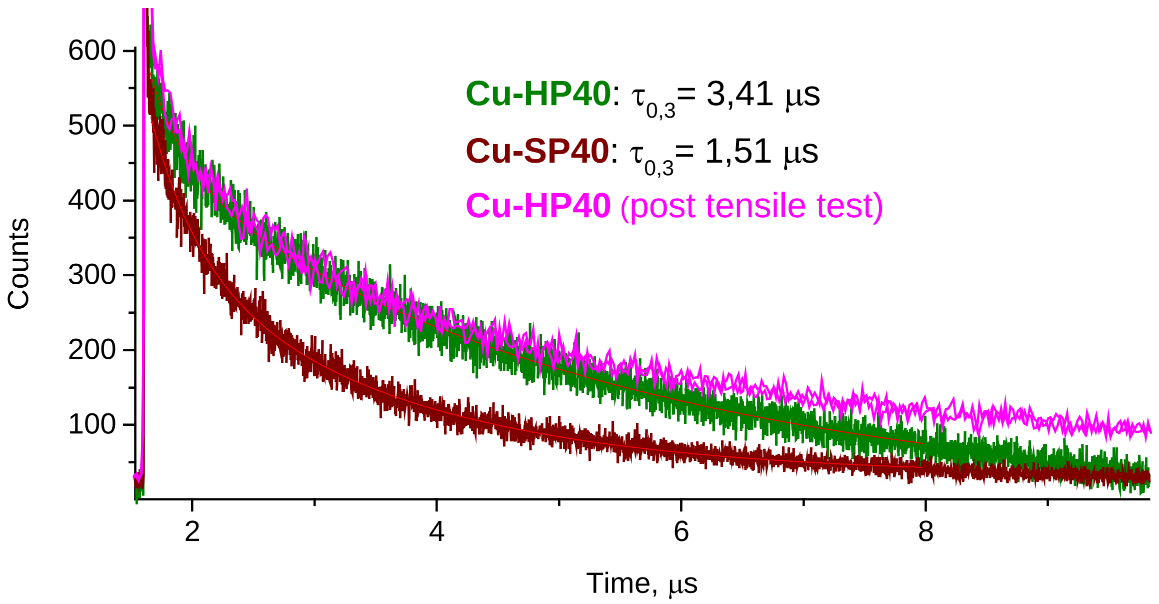

Figure S14. Photoluminescence decay curve of metallated polymer ***films*** **Cu-HP40** and **SP40** measured at 560 nm upon excitation at 404 nm. Overlaid pink lines are **Cu-HP40** samples measured after tensile testing given for comparison. Red lines are double exponential fits given for reference. Indicative lifetime corresponding to the 70% decay given on the graph.

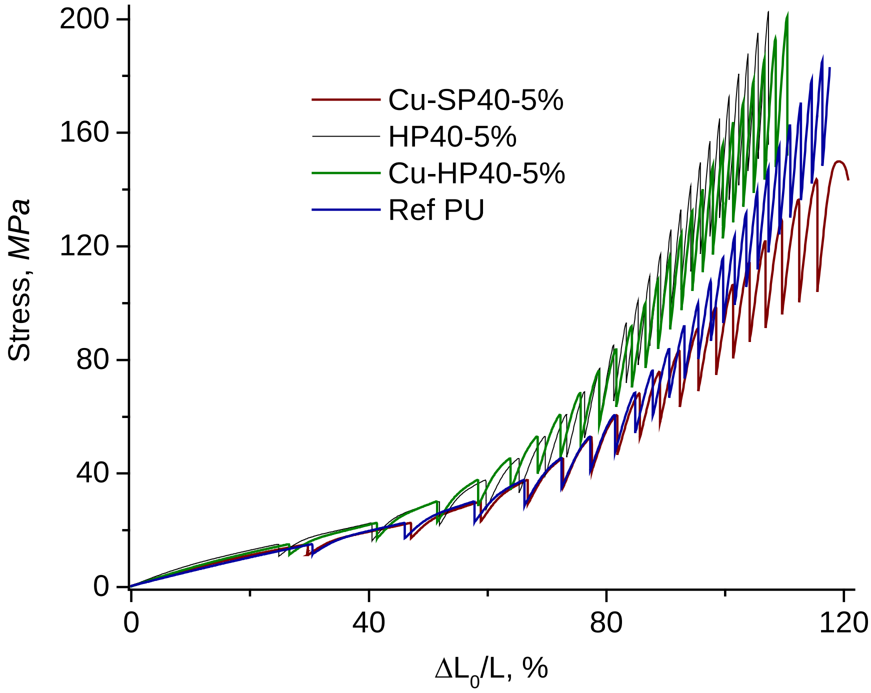

Figure S15. Comparison of stepwise extension test data for Cu-HP40, Cu-SP40, non metallated HP-40 polymer and reference PU with no mechanophore in strain-stress coordinates indicating repeatability of tests. Loading rate 5%L_0/s. L_0 stands for the initial length of sample after prestretch.

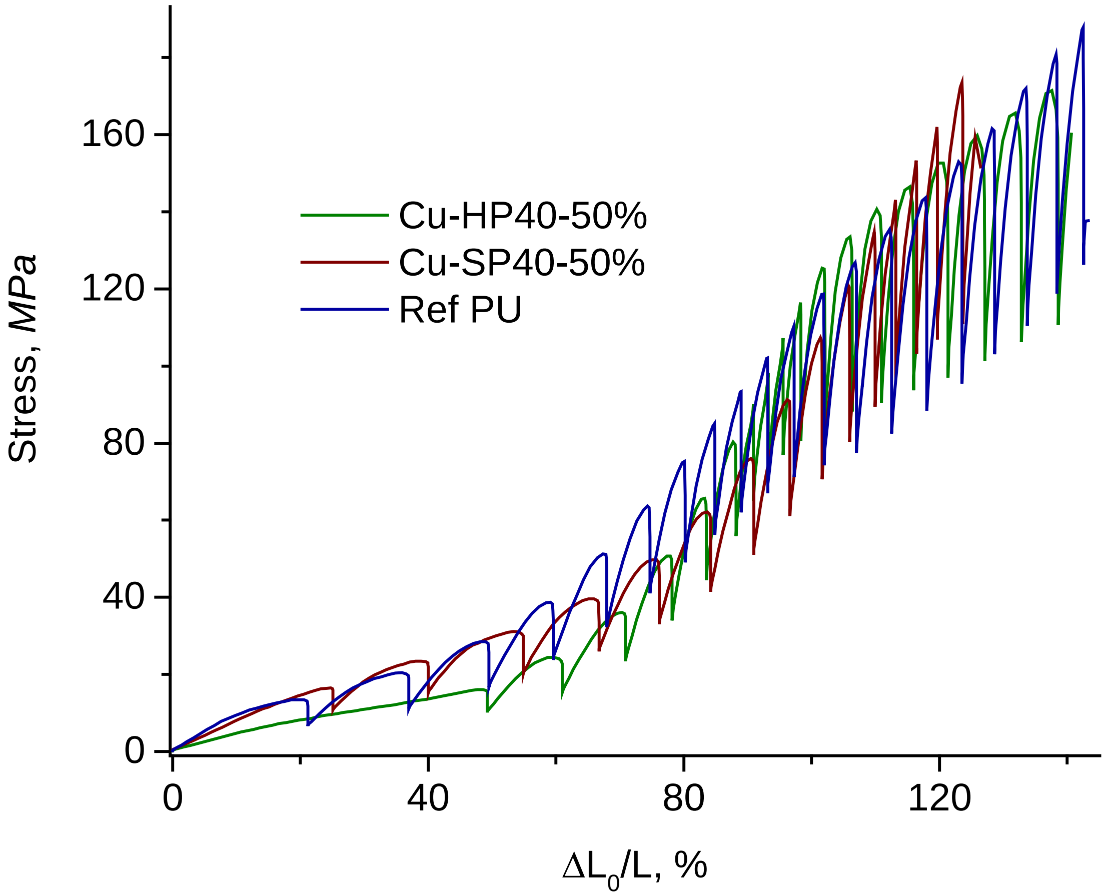

Figure S16 Comparison of stepwise extension test data for Cu-HP40 and Cu-SP40 and reference PU with no mechanophore in strain-stress coordinates. Loading rate 50%L_0/s. L_0 stands for the initial length of sample after prestretch.

S4 Supplementary DSC data

Melting/crystallization behaviour:

Melting behaviour of Cu-HP40 after different loading protocols (fast and slow) was analysed using first heating curves in these samples to find no significant difference in melting enthalpies, although the heat flow curves appeared to be different for these samples. Analysis of first heating curves in general is not straightforward as the sample/pan contact is evolving during these measurements making direct comparison less accurate. However, qualitative analysis of these curves allows concluding that overall melting enthalpy is not significantly affected. See curves below.

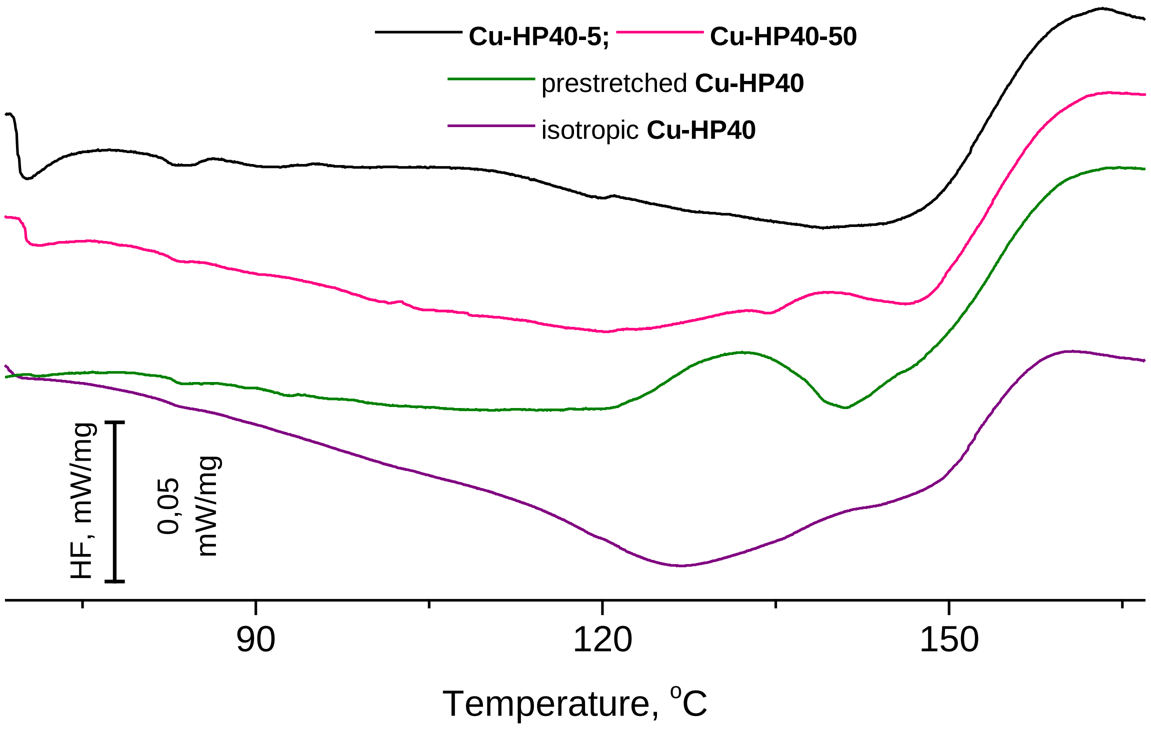

Figure S17 First melting curves for **Cu-HP40** sample set. Curves translated vertically for clarity. Heating rate 10 K/min. Exo up.

To analyse crystallization kinetics of **Cu-HP40** and estimate the timescale of crystallization at room temperature we performed analysis of non-isothermal crystallization kinetics. Namely, samples of **Cu-HP40** were preheated at 175 °C and cooled down with heating rates of 7,5, 10 and 12,5 K/min. The evolved crystallization heat was detected as shown on Figure S18.

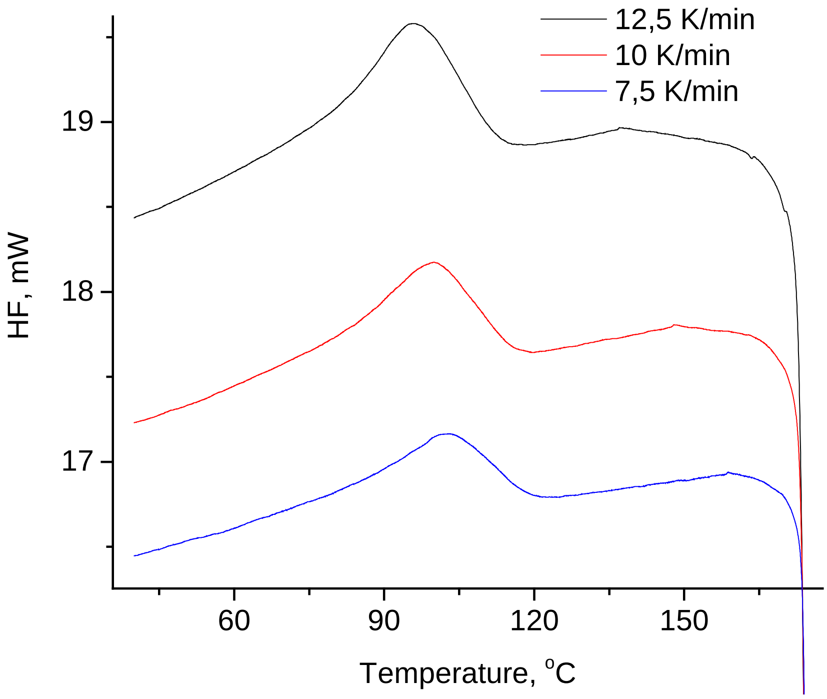

Figure S18 Cooling curves for **Cu-HP40** sample set used in kinetic analysis. Cooling rates indicated on the graph. Exo up.

The data was further analysed using NETZSCH Kinetics Neo software package using model-free approach. Specifically, model-free analysis was performed using Friedman's isoconversional method.[7] Results of this analysis are shown below.

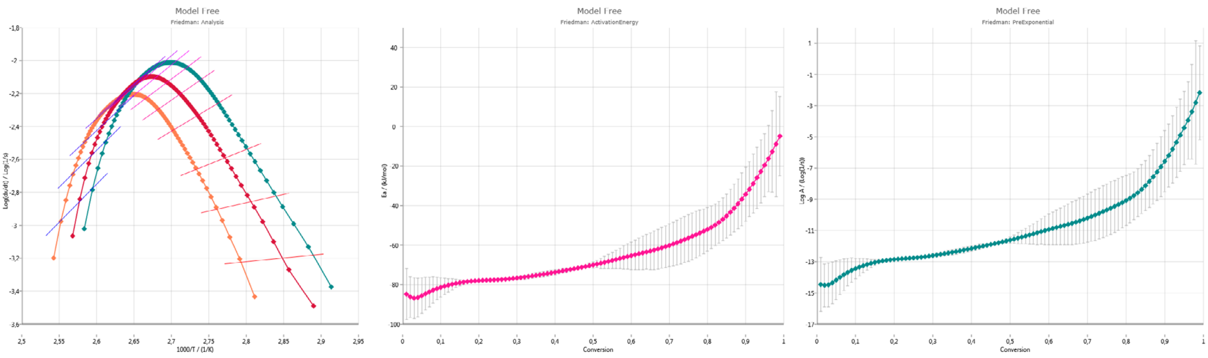

Figure S19 Isoconversional analysis (left) and estimates for activation energy (centre) and pre-exponential factor (right) obtained in model-free analysis. Using estimated values at 50% conversion one can obtain an estimate of the rate constant to be ca. 4.7 s^{-1} at 25°C that places the observed process in a sub-second timescale at this temperature.

To obtain additional data on the timescale of crystallization in **CuHP-40**, predicted isothermal conversion rate was analysed as implemented in NETZSCH Kinetics Neo software. Isothermal reaction progress prediction is given below:

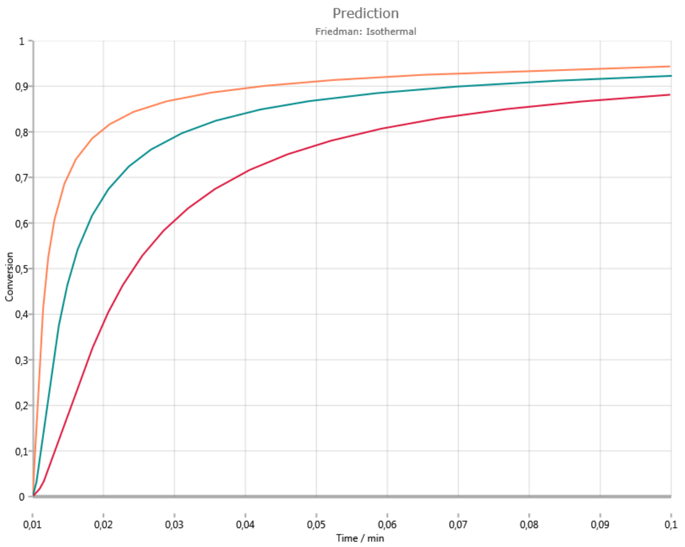

Figure S20 Results of reaction progress modelling using isoconversional methods as implemented in the Kinetics NEO software package. Estimated isothermal conversion vs time plots at 25°C (orange line), 35° (blue) and 45°C (red).

S5 Crystallographic analysis details

Sample **1** for single crystal X-ray diffraction study was grown from acetone/benzene mixture by slow vapour diffusion.

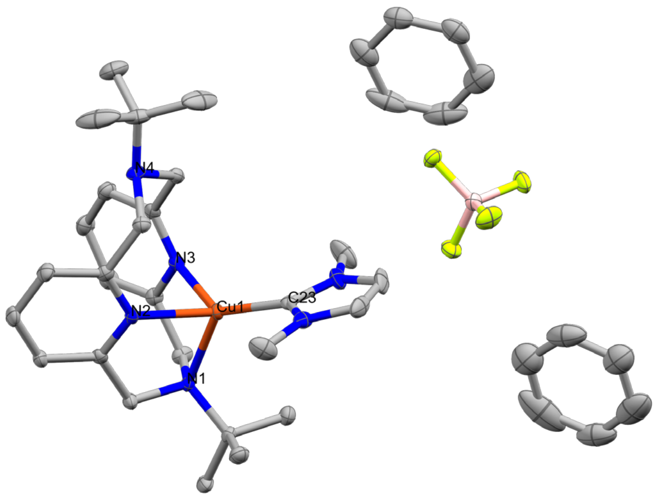

Figure S21 Solid state structure of **1**. Complex **1** co-crystallizes with two benzene molecules that were omitted for clarity in the main body of the manuscript.

Crystallographic data: $C_{27}H_{40}CuN_6(C_6H_6)$, BF_4, $Fw = 755.23$, 0.57×0.10×0.07 mm^3, yellow rod, monoclinic, $P2_1/n$, $a = 9.9728(2)$, $b = 21.1764(4)$, $c = 19.3442(4)$ Å, $\beta = 104.7210(7)°$, $V = 3951.16(14)$ Å^3, $Z = 4$, $D_x = 1.270$ gcm^{-3}, $\mu = 0.61$ mm^{-1}. 31133 reflections were measured by using a Bruker D8-Venture Photon area detector (MoKα radiation, $\lambda = 0.71073$ Å) [8] up to a resolution of $(\sin\theta/\lambda)_{max} = 0.60$ Å^{-1} at a temperature of 100 K. The reflections were corrected for absorption and scaled on the basis of multiple measured reflections by using the SADABS program[8] (0.79–0.85 correction range). 6424 reflections were unique ($R_{int} = 0.041$). Using ShelXle[9], the structures were solved with SHELXS-97[10] by using direct methods and refined with SHELXL-97 on F^2 for all reflections. Non-hydrogen atoms were refined by using anisotropic displacement parameters. The positions of the hydrogen atoms were calculated for idealized positions. With the COSET program,[11] the twin law was determined and then in SHELXL applied. 463 parameters were refined without restraints. $R_1 = 0.035$ for 6424 reflections with $I > 2\sigma(I)$ and $wR_2 = 0.081$ for 7096 reflections, S=1.058, residual electron density was between -0.48 and 0.48 eÅ^{-3}. Geometry calculations and checks for higher symmetry were performed with the PLATON program.[12]

CCDC-1849321 contains the supplementary crystallographic data for this paper. These data can be obtained free of charge from The Cambridge Crystallographic Data Centre via www.ccdc.cam.ac.uk/data_request/cif.

S6 Details of DFT calculations

Density functional theory (DFT) calculations were performed with the hybrid PBE0 exchange-correlation functional[13] using Gaussian 09 D.01 program[14]. Geometry optimizations were performed with all electron 6-31G(d) basis set[15, 16] for all atoms except copper and iodine, for which the LanL2DZ basis set[17, 18] was employed. Van der Waals interactions were described by the dispersion-corrected DFT-D3 (BJ) method.[19] The polarizable continuum model (PCM) with standard parameters for dichloromethane solvent as implemented in Gaussian D.01 was used during the geometry optimization to account for solvent effect. Vibrational analysis was then carried out for all structures at the same level to identify the true nature of the stationary points. No imaginary frequencies were found for local minima, while all transition states exhibited a single imaginary frequency, corresponding to the expected reaction coordinate. Intrinsic reaction coordinate (IRC) approach was employed to confirm the connectivity between the transition states and the corresponding minima. The reaction enthalpies and Gibbs free energies (ΔH and ΔG), and activation enthalpies and Gibbs free energies ($\Delta H^{\ddagger}$ and $\Delta G^{\ddagger}$) were computed with thermal corrections from the normal-mode frequency analysis within the ideal gas approximation at a pressure of 1 atm and temperature of 273 K.

Two isomerization pathways were analysed. Path 1 proceeds through formation of five-coordinate complex and Path 2 proceeds through formation of three-coordinate complex. The reference iodide mechanophore was shown to prefer Path 1 throughout isomerization, therefore the target NHC complex should exhibit similar behaviour to be used as mechanophore. We found a methyl substituted complex $\mathbf{1}^{+}$ (denoted **R** in Figure S22 below) to have appropriate reaction barriers and relative intermediate stabilities nearly identical to those of Cu halide reference. Increase of the bulk on NHC ligand to ^{i}Pr led to a substantial stabilization of three-coordinate Int2 that indicated that isomerization of this complex might involve undesired steps leading to two different intermediate geometries being in competition. Based on this date we opted for the use of N,N’-dimethyl imidazolium motif for mechanophore preparation

Table S1. Reaction enthalpies and Gibbs free energies (ΔH and ΔG), and activation enthalpies and Gibbs free energies ($\Delta H^{\ddagger}$ and $\Delta G^{\ddagger}$) for the isomerization of Cu(I) complexes with different substitutions. Energies are given in kJ mol^{-1}.

Auxiliary ligand	ΔH_1	$\Delta H^{\ddagger}_1$	ΔG_1	$\Delta G^{\ddagger}_1$	ΔH_2	$\Delta H^{\ddagger}_2$	ΔG_2	$\Delta G^{\ddagger}_2$
Iodide (I^-)	9	63	15	**68**	39	101	31	99
NHC_{Me}	-4	56	4	**61**	47	106	35	105
NHC_{iPr}	6	61	**17**	65	46	106	**18**	100

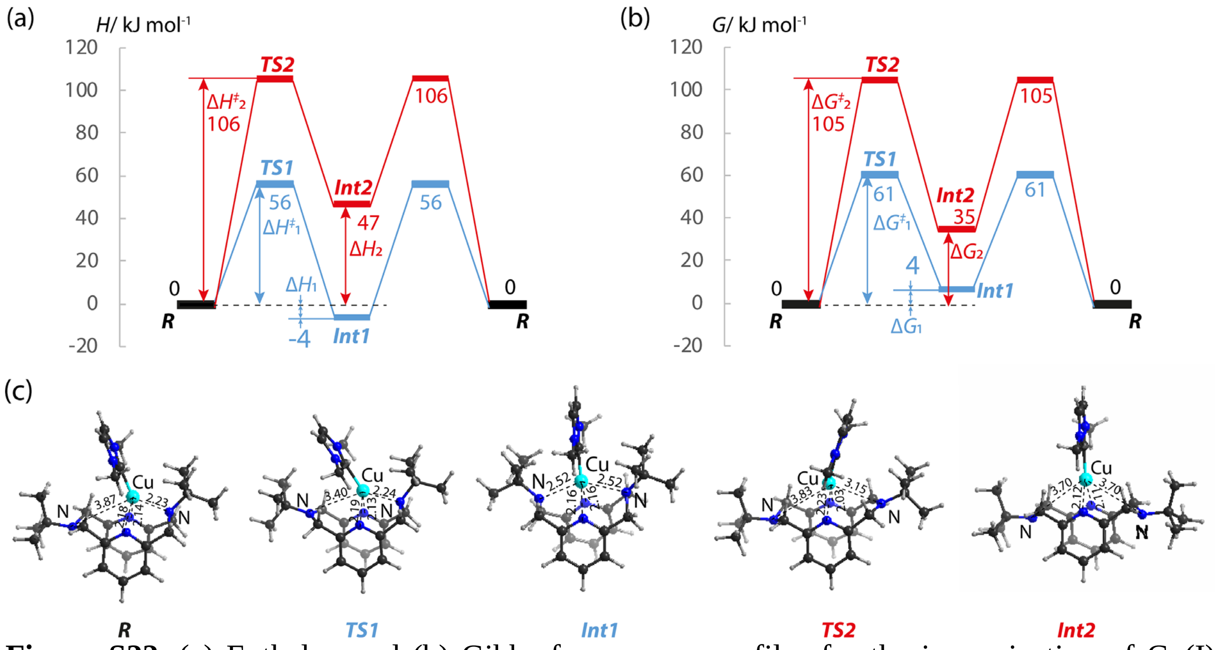

Figure S22. (a) Enthalpy and (b) Gibbs free energy profiles for the isomerization of Cu(I) complexes with methyl substituted NHC ligand. (c) Optimized structures of reaction intermediates (**Int**) and transition states (**TS**). Bond distances are given in Å.

S7 Supplementary X-ray scattering, FTIR, DCS and AFM data

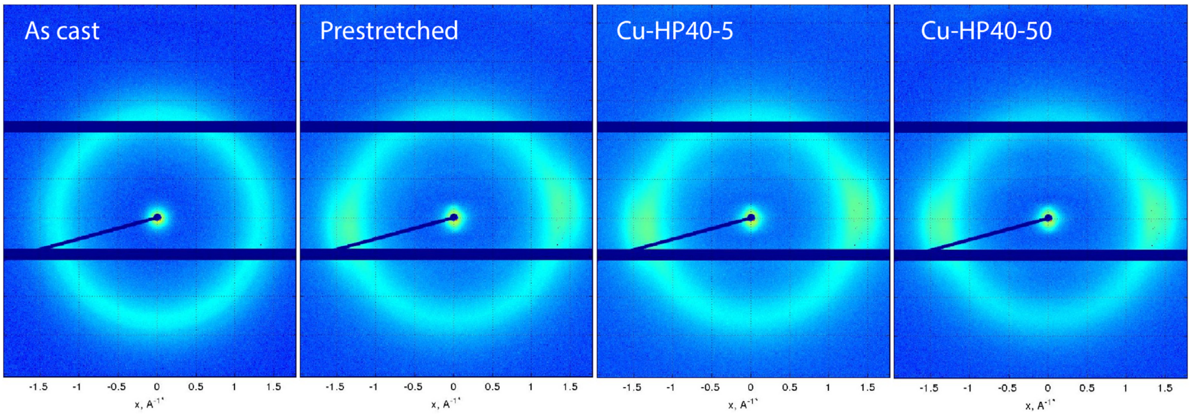

Figure S23 WAXS data for **Cu-HP40** samples discussed in the main text.

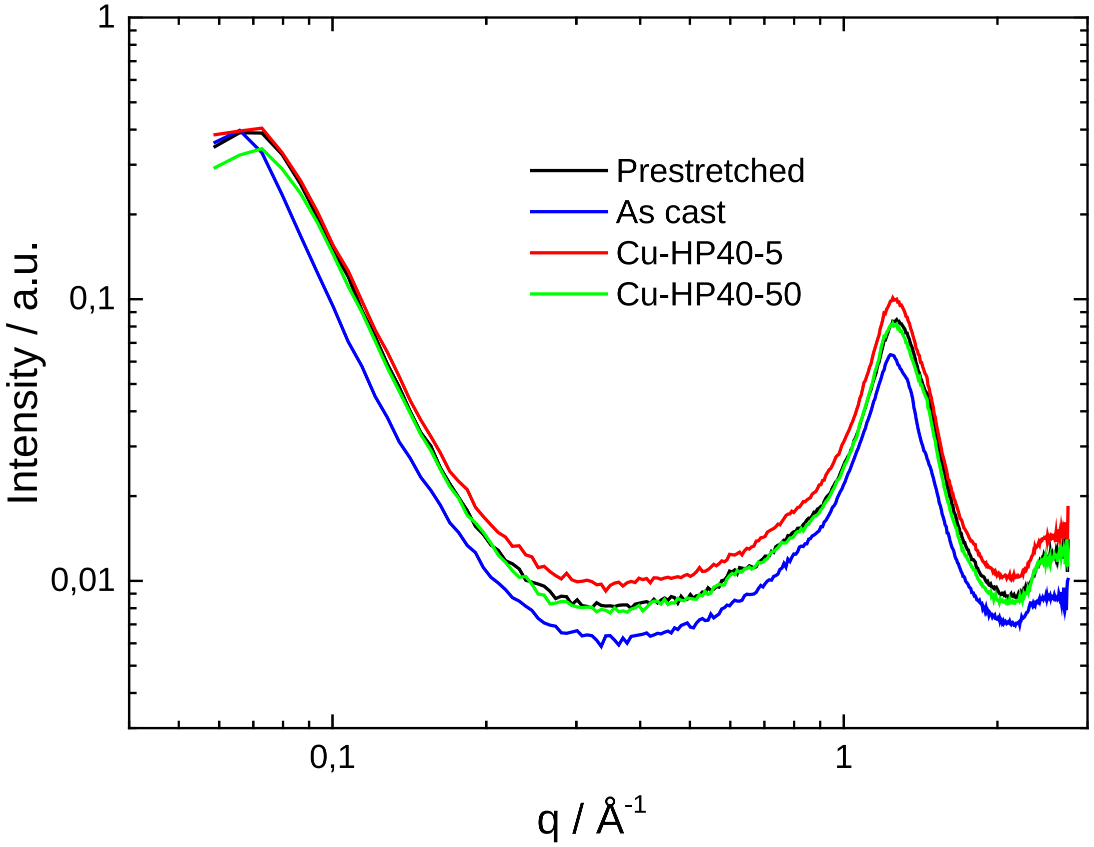

Figure S24 Overlay of integrated WAXS data for **Cu-HP40** samples discussed in the main text indicating no impact of deformation history on the close range order in **Cu-HP40**.

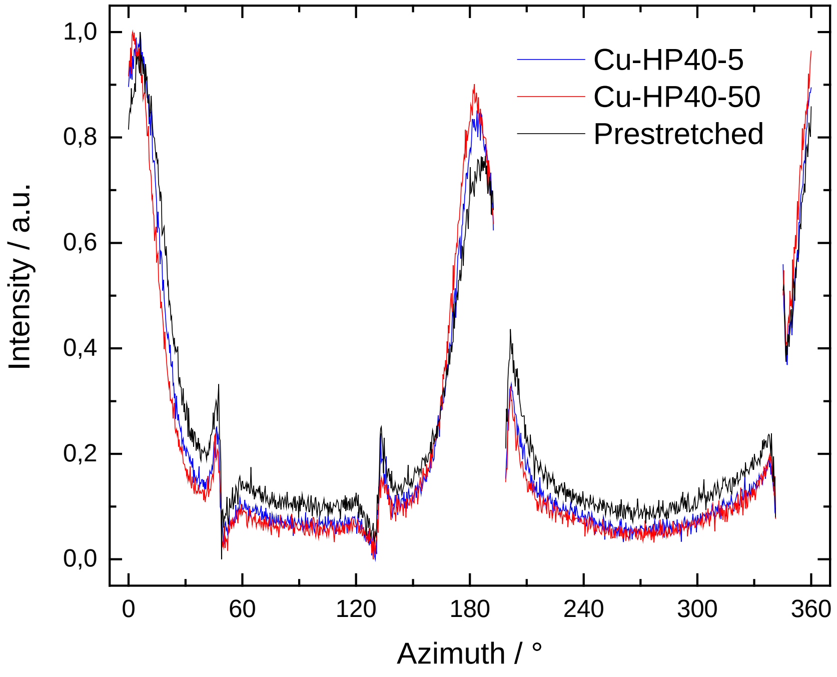

Figure S25 Azimuthal intensity distribution in WAXS plots for non-isotropic **Cu-HP40** samples discussed in the main text.

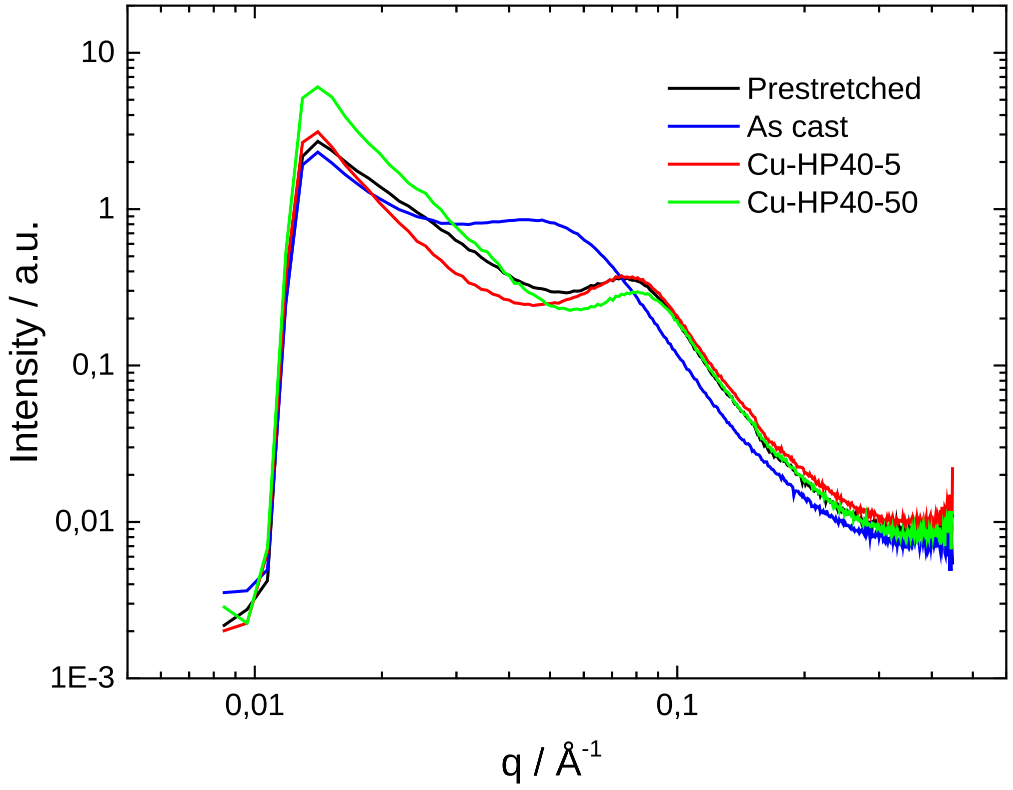

Figure S26 Integrated SAXS data presented in the main text overlaid with that for isotropic “as cast” sample.

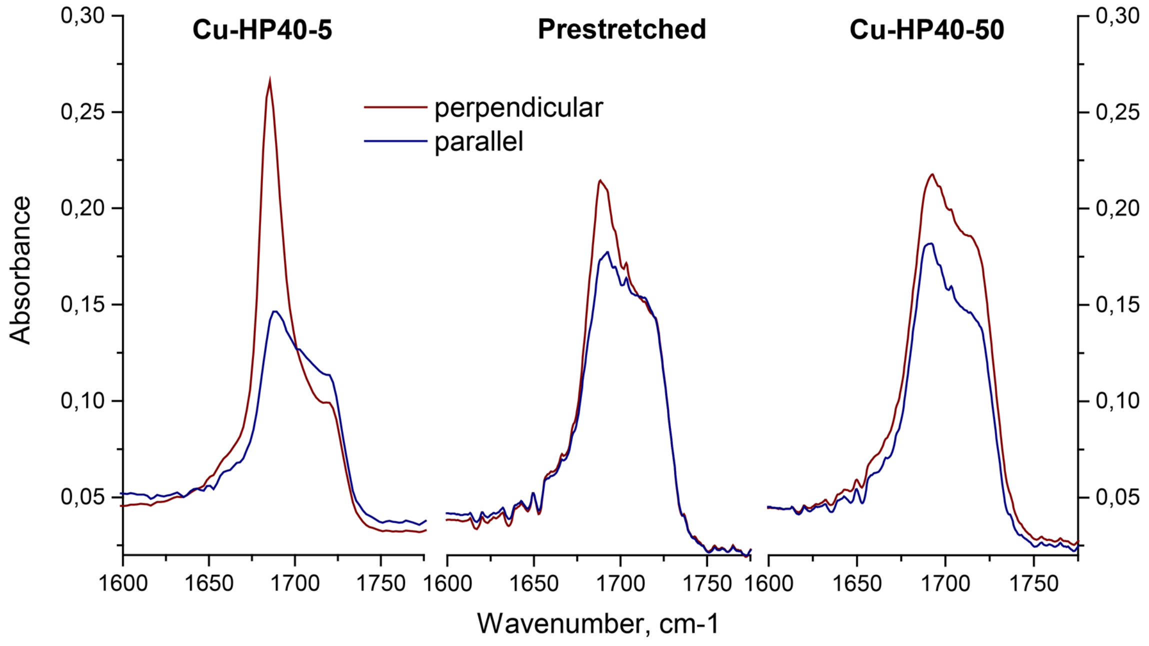

Figure S27 Example of linear dichroism IR data used for dichroic ratio and order parameter calculation.

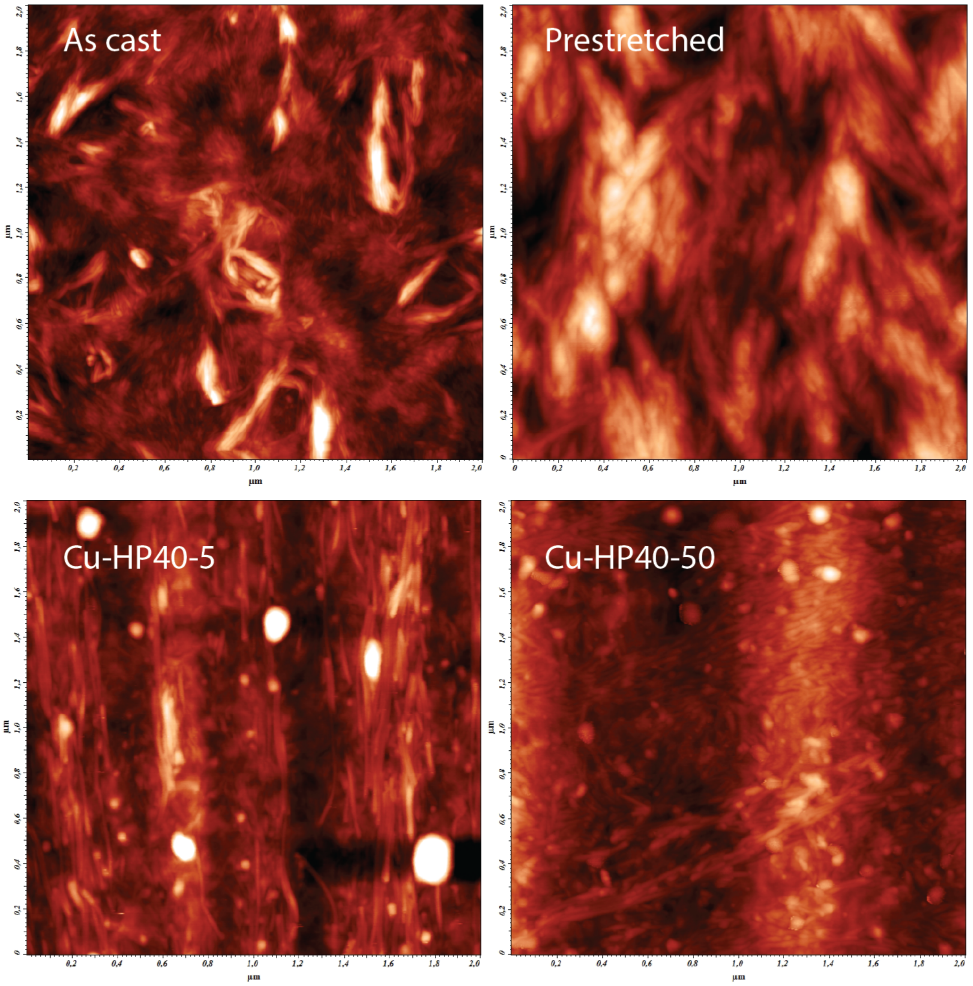

Figure S28 Surface of Cu-HP40 samples examined with AFM.

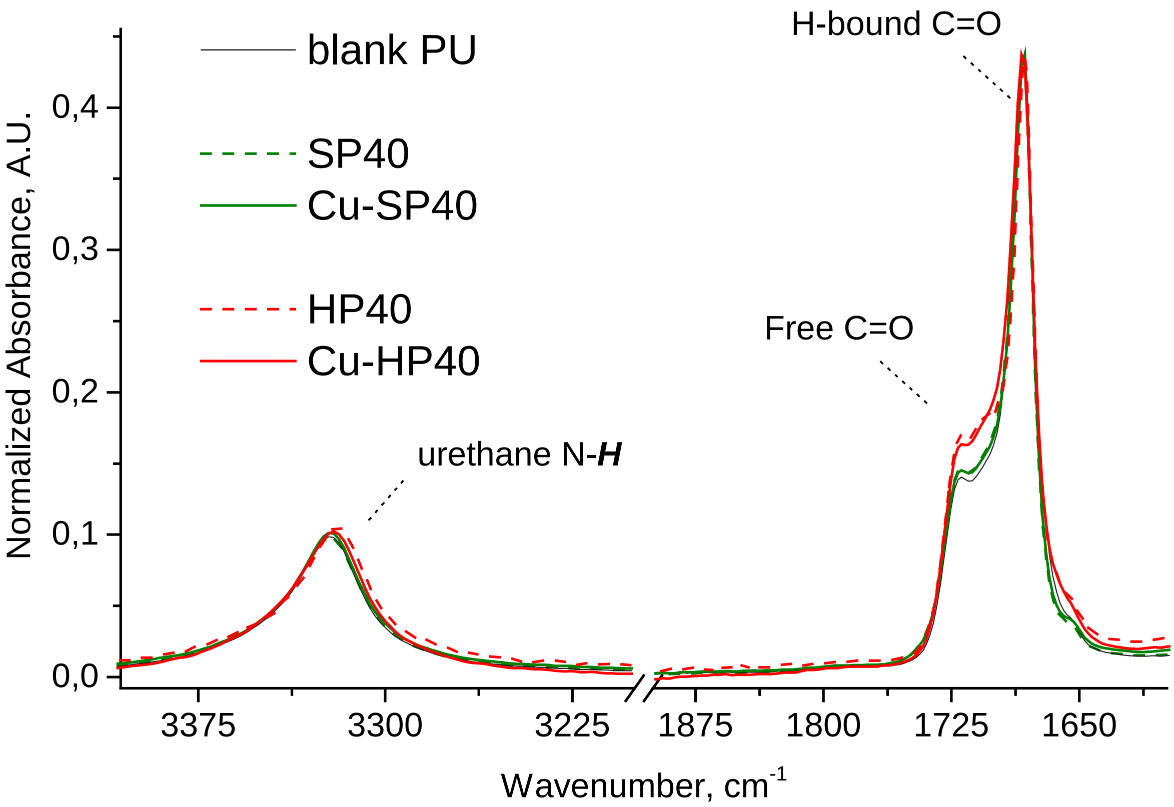

Figure S29 Comparison of the FTIR absorbance for all polymers used in this work with a reference featureless PU sample. Minor increase in free C=O absorbance of HP sample family corresponds to a 2.5% relative increase of the total area.

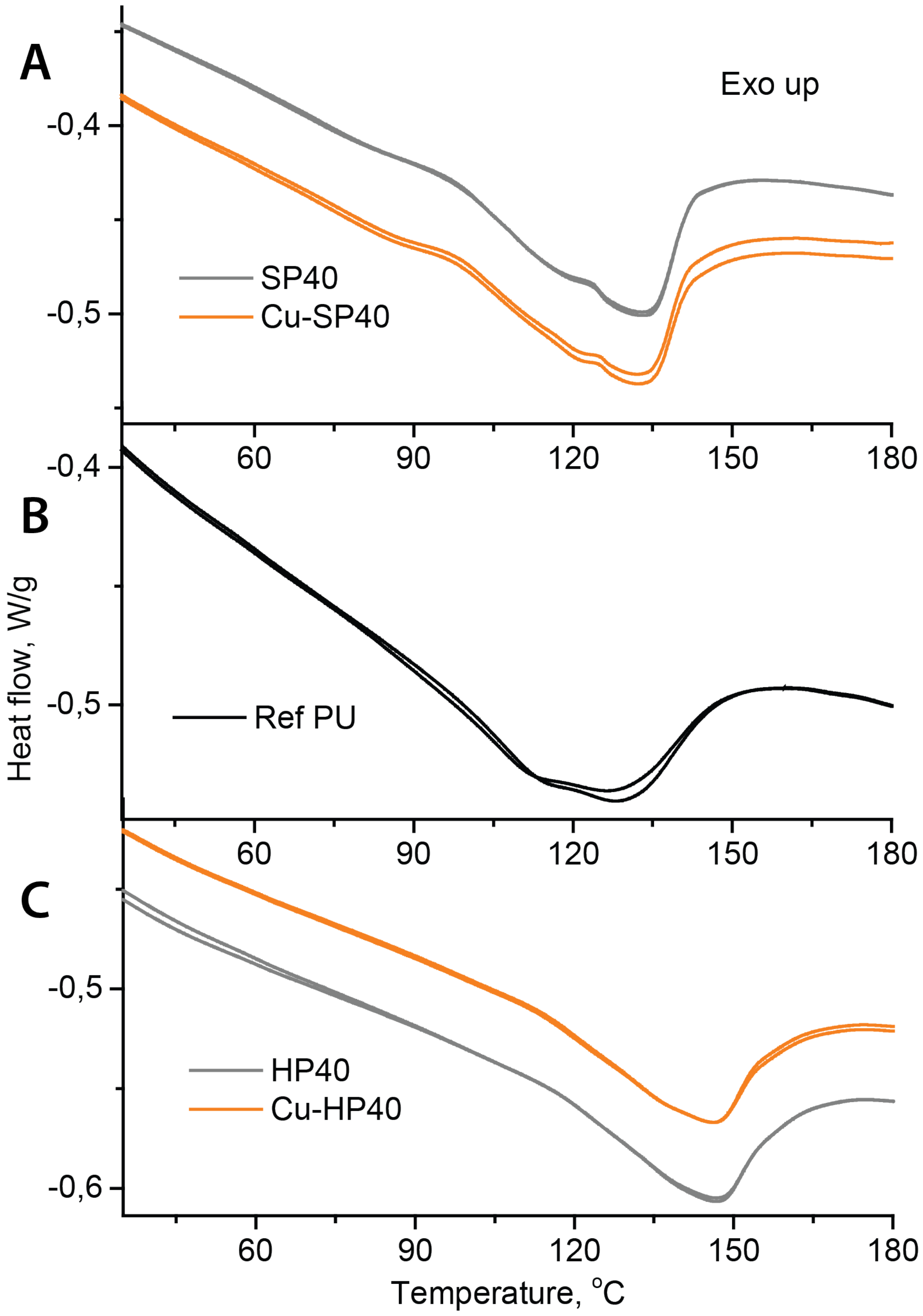

Figure S30 Comparison of the DSC data for 2nd and 3rd melting curves for all PUs discussed in this work. Heating rate 10 K/min.

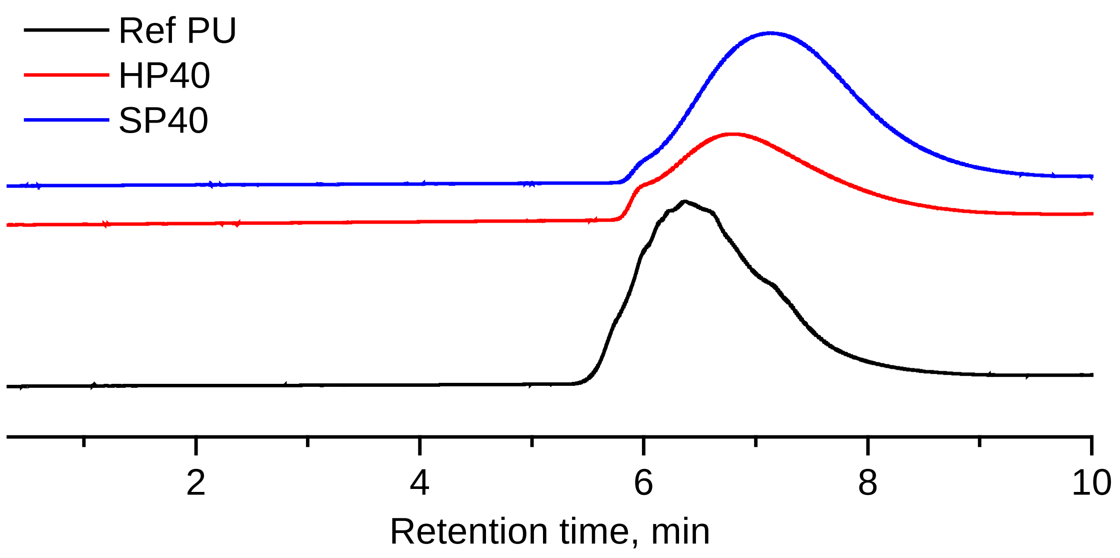

Figure S31 GPC traces of PUs used in this work. Flow rate 1 mL/min, mobile phase DMF-LiBr (0.1M).

References:

1. G. A. Filonenko, J. R. Khusnutdinova, *Adv. Mater.*, 2017, **29**, 1700563
2. R. M. Versteegen, Well-defined thermoplastic elastomers : reversible networks based on hydrogen bonding, DOI: 10.6100/IR562879, PhD Thesis, Eindhoven University of Technology, 2003.
3. E. van Der Heide, O. L. J. van Asselen, G. W. H. Ingenbleek and C. A. J. Putman, *Macromol. Sym.*, 1999, **147**, 127-137.
4. G. Filonenko, R. R. Fayzullin and J. R. Khusnutdinova, *J. Mater. Chem. C*, 2017, **5**, 1638-1645.
5. C.-M. Che, Z.-Y. Li, K.-Y. Wong, C.-K. Poon, T. C. W. Mak and S.-M. Peng, *Polyhedron*, 1994, **13**, 771-776.
6. J. S. Moore and S. I. Stupp, *Macromolecules*, 1990, **23**, 65-70.
7. H. L. Friedman, *J. Polym. Sci. Part C: Polym. Sym.*, 1964, **6**, 183-195.
8. Bruker (2013). APEX2, SAINT and SADABS. Bruker AXS Inc., Madison, Wisconsin,
9. ShelXle is a graphical user interface for SHELXL. B. Hübschle, G. M. Sheldrick and B. Dittrich, *J. Appl. Cryst.*, 2011, **44**, 1281-1284.
10. G. M. Sheldrick, *Acta Cryst.*, 2015, **C71**, 3-8.
11. COSET-Program. P. Boyle, *J. Appl. Cryst.*, 2014, **47**, 467-470.
12. PLATON. A. L. Spek, *Acta Cryst.*, 2009, **D65**, 148–155.
13. C. Adamo and V. Barone, *J. Chem. Phys.*, 1999, **110**, 6158-6170.
14. Gaussian 09 Revision D.01, M. J. Frisch, G. W. Trucks, H. B. Schlegel, G. E. Scuseria, M. A. Robb, J. R. Cheeseman, G. Scalmani, V. Barone, B. Mennucci, G. A. Petersson, H. Nakatsuji, M. Caricato, X. Li, H. P. Hratchian, A. F. Izmaylov, J. Bloino, G. Zheng, J. L. Sonnenberg, M. Hada, M. Ehara, K. Toyota, R. Fukuda, J. Hasegawa, M. Ishida, T. Nakajima, Y. Honda, O. Kitao, H. Nakai, T. Vreven, J. A. Montgomery Jr., J. E. Peralta, F. Ogliaro, M. Bearpark, J. J. Heyd, E. Brothers, K. N. Kudin, V. N. Staroverov, R. Kobayashi, J. Normand, K. Raghavachari, A. Rendell, J. C. Burant, S. S. Iyengar, J. Tomasi, M. Cossi, N. Rega, N. J. Millam, M. Klene, J. E. Knox, J. B. Cross, V. Bakken, C. Adamo, J. Jaramillo, R. Gomperts, R. E. Stratmann, O. Yazyev, A. J. Austin, R. Cammi, C. Pomelli, J. W. Ochterski, R. L. Martin, K. Morokuma, V. G. Zakrzewski, G. A. Voth, P. Salvador, J. J. Dannenberg, S. Dapprich, A. D. Daniels, Ö. Farkas, J. B. Foresman, J. V. Ortiz, J. Cioslowski and D. J. Fox, Gaussian, Inc., Wallingford CT, 2009
15. P. C. Hariharan and J. A. Pople, *Theor. Chim. Acta.*, 1973, **28**, 213-222.
16. M. J. Frisch, J. A. Pople and J. S. Binkley, *J. Chem. Phys.*, 1984, **80**, 3265-3269.
17. P. J. Hay and W. R. Wadt, J. *Chem. Phys.*, 1985, **82**, 299-310.
18. P. J. Hay and W. R. Wadt, J. *Chem. Phys.*, 1985, **82**, 270-283.
19. G. Stefan, E. Stephan and G. Lars, J. *Comput. Chem.*, 2011, **32**, 1456-1465.
